# Health equity and public acceptance of large language models in healthcare in China: A national population-based survey

**DOI:** 10.1371/journal.pdig.0001555

**Published:** 2026-07-30

**Authors:** Jiaying Li, Helen Yue Lai Chan, Zengjie Ye, Xiang Qi, Wai Tong Chien, Ka Ming Chow

**Affiliations:** 1 The Nethersole School of Nursing, Faculty of Medicine, The Chinese University of Hong Kong, Hong Kong SAR, China; 2 School of Nursing, Guangzhou Medical University, Guangzhou, Guangdong, China; 3 NYU Rory Meyers College of Nursing, New York University, New York, New York, United States of America; National Tsing Hua University, TAIWAN

## Abstract

Large language models (LLMs) are entering healthcare, but their ability to improve access depends on public willingness to use them. If acceptance is socially patterned, deployment may widen existing inequities. We quantified public acceptance of LLMs in healthcare and examined its bio-psycho-social correlates in China. We conducted a representative, multistage stratified survey of adults aged 18 years or older across 150 Chinese cities between June and September 2024. After a standardized description of LLM capabilities, participants rated acceptance of LLMs in healthcare on a 0–100 scale. Survey-weighted regression identified correlates of acceptance, and Classification and Regression Tree (CART) analysis identified profiles associated with non-acceptance. Among 35,861 respondents, weighted mean acceptance was 64.3/100 (95% CI 63.9–64.6). Acceptance was lower among respondents with chronic conditions than in the overall sample (62.4 vs 64.3) and declined with age. Higher acceptance was associated with greater perceived social status, prior digital health use, self-efficacy, stronger family-neighbour relationships, and higher eHealth literacy (β_std = 0.06 to 0.19), whereas lower acceptance was associated with older age, adverse social and developmental exposures, severe ADHD symptoms, social loneliness, and financial strain (β_std = -0.04 to -0.09). In CART analysis, lack of prior digital health use defined the largest non-acceptor group, comprising 62% of the sample. Among those with prior digital experience, lower social support and lower childhood socioeconomic status remained important markers of non-acceptance. In the test set, the tree showed modest discrimination (weighted AUC 0.62, 95% CI 0.61-0.64), with high specificity (0.94) and low sensitivity (0.19). Public acceptance of LLMs in healthcare in China was moderate but unequal. Lower acceptance among older adults, people with chronic conditions, and those with fewer social and digital resources suggests socially patterned uptake. Equity-oriented implementation strategies are needed so LLM integration does not preferentially benefit advantaged groups.

## Author summary

Large language models are rapidly entering healthcare, but their value will depend on whether the public is willing to use them. We examined public acceptance of these tools in China in a national survey of 35,861 adults. The mean acceptance score was 64.3 out of 100, suggesting moderate but far from universal acceptance. Overall, acceptance was moderate rather than high. We found that willingness to use large language models in healthcare was not evenly distributed across the population. Older adults, people with chronic conditions, and those with fewer social and digital resources were less accepting. In contrast, people with stronger confidence, better digital experience, and greater social support tended to be more accepting. We also found that lack of prior digital health use was the strongest marker of non-acceptance. These findings matter because new health technologies do not automatically reduce inequality; they can also deepen it if they mainly benefit people who are already better resourced. Our study shows that the future implementation of large language models in healthcare should pay close attention to fairness, accessibility, and support for groups that may otherwise be left behind.

## 1. Introduction

Large language models (LLMs), a class of generative artificial intelligence (AI) systems trained on large-scale text data, are increasingly being incorporated into healthcare. Their applications now span multiple stages of care delivery, including summarizing unstructured clinical records, assisting evidence synthesis for clinicians, and supporting patient-facing functions such as health information retrieval, self-triage, medication guidance, and mental health support [1–4]. Reflecting this rapid expansion, the World Health Organization has highlighted generative AI as an emerging resource with the potential to reshape clinical care and public health systems [5]. However, the successful integration of LLMs into healthcare will depend not only on technical performance, such as accuracy and safety, but also on whether the public is willing to accept and engage with these tools.

Public acceptance matters because LLMs are increasingly positioned as direct or semi-direct interfaces between individuals and health information or services. In this study, we define acceptance as self-reported willingness or readiness to use LLMs in healthcare after receiving a standardized description. This construct is distinct from trust, perceived usefulness, and general attitudes, and reflects a proximal implementation outcome indicating public readiness to engage with LLM-enabled healthcare. Although these LLM-enabled healthcare may improve access to timely and scalable support, its diffusion may be uneven. Reviews of patient perspectives suggest that people may value the efficiency and convenience of broader AI-enabled health tools while remaining concerned about harmful advice, lack of professional oversight, data privacy, and the potential erosion of patient-provider relationships [6,7]. More broadly, recent national surveys in high-income settings have shown persistent public skepticism about the responsible use of AI in healthcare more generally [8]. However, much of this evidence relates to AI in general rather than LLMs specifically, and population-based evidence on public acceptance of LLM-enabled healthcare remains limited. Because LLMs interact directly with users, public acceptance is a critical determinant of implementation. Failure to understand socially patterned acceptance may widen digital health inequities, reduce effective engagement with LLM-supported services, and lead policymakers to invest in technologies without adequate support for groups less ready to use them.

Existing research on acceptance has also been narrow in scope. Most studies have focused on technology-centred determinants, such as usability, perceived usefulness, or anthropomorphic design features [9]. These factors explain how users interact with a tool, but fail to explain the deeper human determinants of why they engage. Existing evidence hints at a digital divide: younger individuals, men [10,11], those with higher education [12], and those with higher eHealth literacy [13] show significantly higher uptake. Yet prior studies have generally relied on relatively small or non-representative samples and limited sets of predictors. As a result, little is known about how broader bio-psycho-social factors, including social position, life adversity, social connectedness, personality, health status, and prior digital experience, are associated with public acceptance of LLMs in healthcare, particularly outside predominantly Western settings.

China provides an important setting in which to examine these questions. As one of the world’s most rapidly digitizing health environments, it offers a valuable context for assessing public acceptance of LLMs at scale and for identifying groups who may be less likely to engage with these technologies. While qualitative approaches are highly valuable for unpacking the nuances of human-computer interaction and interface preferences, quantifying the systemic digital divide requires population-level epidemiological profiling. Therefore, a large-scale, quantitative survey is methodologically necessary to systematically capture the distribution of acceptance and mathematically model the structural, social, and psychological determinants across a massive, heterogeneous population.

In this study, we used data from a large, population-based, multistage stratified survey of adults in China to: (1) quantify acceptance of LLMs in healthcare; (2) examine independent associations between acceptance and 139 candidate predictors spanning sociodemographic, psychosocial, lifestyle, physical health, and mental health domains; and (3) identify profiles associated with non-acceptance using classification-tree analysis. By characterizing how acceptance varies across population subgroups, this study aims to inform more equitable implementation of LLM-enabled healthcare.

## 2. Methods

### 2.1 Study design and participants

This study is a secondary analysis of data from a national cross-sectional survey in China that was originally designed to assess the psychological and behavioral health status. The survey used a multistage stratified sampling strategy. Participants were recruited through field-based on-site recruitment. Trained surveyors visited selected survey sites, screened potential participants for eligibility, obtained electronic informed consent, and administered the electronic questionnaire. To ensure representativeness, we selected 150 cities across all 31 provincial-level administrative regions. Sampling was carried out in four stages: district/county (n = 202), township/street (n = 390), and community/village (n = 800), ensuring equal probability at each level. Eligible participants were Chinese nationals aged ≥18 years who had resided in the area for ≥11 months of the past year. We excluded individuals with psychosis or cognitive impairment, or those unable to provide informed consent.

### 2.2 Data collection

Between 23 June and 29 September 2024, trained surveyors conducted on-site recruitment and administered an electronic questionnaire. Prior to enrollment, surveyors detailed the study’s purpose, procedures, and the voluntary nature of participation, ensuring that respondents understood their right to decline. All participants provided explicit electronic informed consent. To protect confidentiality, survey data were securely stored, de-identified prior to analysis, and reported exclusively in aggregate. To ensure data quality, responses underwent logic verification and automated quality checks; submissions with >20% missing data or completion times <300 seconds were rejected. Of 38,793 questionnaires distributed, 38,424 (99.0%) were returned. Following the strict exclusion criteria for age violations, non-consent, or rapid completion, 35,861 valid records were retained for analysis (S1 Fig).

### 2.3 Measures of acceptance of LLMs in healthcare

The primary outcome was acceptance of LLMs in healthcare, measured on a visual analog scale from 0 (completely unacceptable) to 100 (completely acceptable). To reduce variation in baseline understanding of emerging AI technologies, all participants were presented with a standardized plain-language description of LLMs before rating their acceptance. The standardized text stated: “Large language models (LLMs) are a type of deep learning model trained on massive amounts of text data to generate human-like responses. In healthcare, LLMs can affect patients’ health by providing answers to medical questions, assisting with diagnosis and treatment plans, and automating healthcare administrative tasks.” Following this description, participants were asked: “What is your acceptance of the application of large language models in healthcare?”.

### 2.4 Measures of individual-level factors

We assessed 139 candidate predictors across seven domains (comprehensive variable definitions, full names for all scale abbreviations, and scale reliability metrics are provided in S1 Table

#### 2.4.1 Sociodemographics (26 variables).

We assessed demographic factors (age, sex, gender identity, sexual orientation, handedness, ethnicity, religion); SES (education, occupation, income, city tier, hukou [household registration] status, perceived social status); household context (relationship status, family structure, siblings, living arrangement, housing characteristics [area and structure], assets and debt, residential context, duration of residence); and healthcare access (insurance, enrollment site, payment difficulty).

#### 2.4.2 Life history and adversity (30 variables).

We captured 14 past-year stressors (e.g., bereavement, unemployment, legal issues) and 15 adverse childhood experiences (ACEs) (covering various forms of abuse, neglect, household dysfunction, and financial hardship), plus perceived childhood SES.

#### 2.4.3 Personality and self-efficacy (7 variables).

We measured personality (five dimensions assessed via the Mini Big-Five inventory), general self-efficacy (NGSES-3), and narcissistic admiration/rivalry (NARQ-8).

#### 2.4.4 Literacy, empowerment, and social capital (15 variables).

Measures included social capital (perceived social support via PSSS-12, family-neighbor relationships, confidant availability, tangible help, social isolation, loneliness, and social connection); family dynamics (family health via FHS-10, family communication via FCS-6); competency (eHealth literacy via eHEALS-8; general literacy via HLS-SF16; antibiotic knowledge; implicit health beliefs; eating self-regulation via SREBQ-5); and prior digital health use.

#### 2.4.5 Lifestyle factors (14 variables).

We recorded substance use (tobacco, alcohol); physical activity (IPAQ-7; MET categories); sleep (chronotype, duration, quality, snoring, daytime sleepiness); dietary habits (such as the habit of adding extra salt to food); and cultural engagement (music, arts, dance, other arts).

#### 2.4.6 Physical health (37 variables).

Respondents were asked to self-report biometrics (body mass index using Asian-specific cut-points); health status (EQ-5D-5L; 14 physician-diagnosed chronic diseases; 14 injury types); and immunization history (number of COVID-19 infections; vaccination status for human papillomavirus, influenza, shingles, hepatitis B virus, COVID-19, and none).

#### 2.4.7 Mental health (10 variables).

We screened psychological conditions and symptoms using validated scales: depression (PHQ-9), anxiety (GAD-3), stress (PSS-4), attention-deficit/hyperactivity disorder (ADHD) traits (ASRS-6), and work burnout (CCBI-7). Psychosocial traits included cyberchondria (SCS-5), rest intolerance (RSS-8), maladaptive daydreaming (MDS-5), social media addiction (BSMAS-6), and perceived personal existence (single-item measure of meaning in life).

### 2.5 Data analysis

Statistical analyses were performed using R (version 4.1.1). We employed a three-stage analytic framework: (1) population-weighted estimation; (2) dual-strategy predictor identification (hierarchical regression validated by Elastic-Net); and (3) decision-tree classification.

First, to enhance national representativeness, we applied post-stratification raking weights so that our sample aligned with the age and sex distribution of the 2020 Chinese census. We calculated weighted prevalence and means with 95% confidence intervals (CIs) by province, age-sex strata, single-year age (locally estimated scatterplot smoothing [LOESS]-smoothed by sex) [14], and chronic conditions.

Second, to isolate independent associations while managing high dimensionality, we compared two modeling approaches: survey-weighted hierarchical linear regression and Elastic-Net regularization. Because acceptance of LLMs in healthcare may be associated with multiple social, developmental, psychological, behavioral, and health-related factors, we selected 139 candidate predictors a priori within a broad biopsychosocial framework. To improve interpretability and reduce the risk of overfitting, predictors were organized into seven prespecified conceptual blocks rather than entered as an unstructured set of variables. We entered predictors in seven blocks following a prespecified conceptual order: sociodemographics, adversity, personality and self-efficacy, literacy, empowerment, and social capital, lifestyle, physical health, and mental health. Within each block, we applied Benjamini-Hochberg correction to control the false discovery rate, carrying forward only significant terms to subsequent blocks to reduce model size and limit overinterpretation of unstable associations. Multicollinearity was monitored (variance inflation factor [VIF] cutoff >3) [15]. Then, to validate the hierarchical selection, we fitted an Elastic-Net model with all 139 candidate predictors (standardized), with 10-fold cross-validation (α = 0.50) to identify the optimal penalty parameter (λ_min_) that minimized the mean squared error. We reported overlap statistics to quantify concordance between the hierarchical and regularized models. Individual coefficients were interpreted cautiously, with emphasis on broader domain-level patterns rather than isolated causal effects.

Third, to identify distinct profiles of non-acceptance, we dichotomized acceptance (≥80 defined as acceptors vs. < 80 as non-acceptors). The weighted sample was split 80/20 into training and test sets (outcome-stratified). We trained a Gini-based classification and regression tree (CART) model incorporating sampling weights [16], optimizing the complexity parameter via the elbow method. We evaluated the model on the test set by calculating a survey-weighted area under the receiver operating characteristic curve (AUC) with 1,000 bootstrapped CIs, alongside sensitivity, specificity, and positive and negative predictive values.

## 3. Results

### 3.1 Population characteristics

The weighted sample comprised 35,861 adults (mean age 47.5 ± 16.7 years; 50.4% men; 93.1% Han ethnicity). Most participants (82%) had completed secondary education, and 72% resided in urban areas. Full characteristics are detailed in Table 1.

**Table 1:** Sociodemographic and health characteristics of participants before and after weighting (n = 35,861).

Characteristic	Categories	n (%)/mean (SD)	
		Unweighted (n = 35,861)	Weighted* (n = 35,861)
Age (years), mean (SD)	–	34.88 (16.48)	47.50 (16.74)
Social status score^†^, mean (SD)	–	4.00 (1.35)	4.11 (1.38)
Sex	Male	15,428 (43.0)	18,084 (50.4)
	Female	20,275 (56.5)	17,639 (49.2)
	Other	158 (0.4)	138 (0.4)
Race/ethnicity	Han ethnicity	32,228 (89.9)	33,381 (93.1)
	Other	3,633 (10.1)	2,480 (6.9)
Religion (any)	No	32,635 (91.0)	32,134 (89.6)
	Yes	3,226 (9.0)	3,727 (10.4)
Education	Primary or below	3,211 (9.0)	6,452 (18.0)
	Secondary	11,189 (31.2)	13,182 (36.8)
	Undergraduate	19,862 (55.4)	14,416 (40.2)
	Graduate	1,599 (4.5)	1,812 (5.1)
Employment status	Employed	10,449 (29.1)	13,889 (38.7)
	Retired	2,922 (8.1)	6,677 (18.6)
	Self-employed	5,781 (16.1)	8,615 (24.0)
	Student	14,870 (41.5)	3,735 (10.4)
	Unemployed	1,839 (5.1)	2,945 (8.2)
Per capita monthly income (CNY)	≤1000	2,068 (5.8)	1,888 (5.3)
	1001-2000	3,053 (8.5)	2,946 (8.2)
	2001-3000	4,461 (12.4)	4,601 (12.8)
	3001-4000	5,362 (15.0)	5,547 (15.5)
	4001-5000	4,789 (13.4)	4,974 (13.9)
	5001-6000	4,601 (12.8)	4,689 (13.1)
	6001-9000	4,591 (12.8)	4,638 (12.9)
	9001-12000	2,945 (8.2)	2,809 (7.8)
	12001-15000	1,693 (4.7)	1,610 (4.5)
	≥15001	2,298 (6.4)	2,160 (6.0)
City tier (Hukou location)	First-tier city	2,052 (5.7)	2,620 (7.3)
	New first-tier city	4,645 (13.0)	5,190 (14.5)
	Other city tiers	29,164 (81.3)	28,051 (78.2)
Residence in last 3 months	Urban	27,062 (75.5)	26,005 (72.5)
	Rural	8,799 (24.5)	9,856 (27.5)
Household registration	Urban	16,728 (46.6)	17,883 (49.9)
	Rural	19,133 (53.4)	17,978 (50.1)
Medical insurance	None	2,344 (6.5)	1,661 (4.6)
	Government-funded only	2,779 (7.7)	2,336 (6.5)
	Urban resident only	14,742 (41.1)	13,021 (36.3)
	Employee only	5,528 (15.4)	7,604 (21.2)
	New rural cooperative only	3,870 (10.8)	4,874 (13.6)
	Commercial only	547 (1.5)	475 (1.3)
	Two types	4,858 (13.5)	4,799 (13.4)
	More than two types	1,193 (3.3)	1,092 (3.0)
Diagnosed health conditions	Hypertension	2,816 (7.9)	5,687 (15.9)
	Diabetes	993 (2.8)	1,990 (5.6)
	Hyperlipidemia	791 (2.2)	1,509 (4.2)
	Coronary heart disease	453 (1.3)	939 (2.6)
	Stroke	110 (0.3)	202 (0.6)
	Respiratory disease	511 (1.4)	770 (2.1)
	Urinary disease	253 (0.7)	403 (1.1)
	Digestive Disease	814 (2.3)	1,094 (3.1)
	Osteoporosis	798 (2.2)	1,591 (4.4)
	Arthritis	1060 (3.0)	1,944 (5.4)
	Tumor	196 (0.5)	292 (0.8)
	Obesity	6,156 (17.4)	7,585 (21.5)
	Depression	8,192 (22.8)	6,891 (19.2)
	Anxiety	6,129 (17.1)	5,160 (14.4)
	Rare disease	69 (0.2)	71 (0.2)

* Weights were applied based on the 2020 Chinese Census age-sex distribution.

†Measured on a 1–7 scale where 7 represents the highest social standing.

***Note***: SD, standard deviation; Hukou, household registration; first-tier and new first-tier, city-tier classifications in China reflecting level of urban development and economic activity.

### 3.2 Weighted acceptance by region, age-sex, and chronic condition

Weighted overall acceptance was 64.3 (95% CI 63.9–64.6). A distinct regional gradient emerged, in which scores peaked in the Northeast (Jilin 72.3; Liaoning 71.9) and affluent coastal provinces (Jiangsu 69.4) but dropped in autonomous regions (Inner Mongolia 55.5; Tibet 57.1). Among the major metropolitan regions, Shanghai (67.2) and Guangdong (67.2) outpaced Beijing (63.0) (S2 Table).

**Fig 1:**
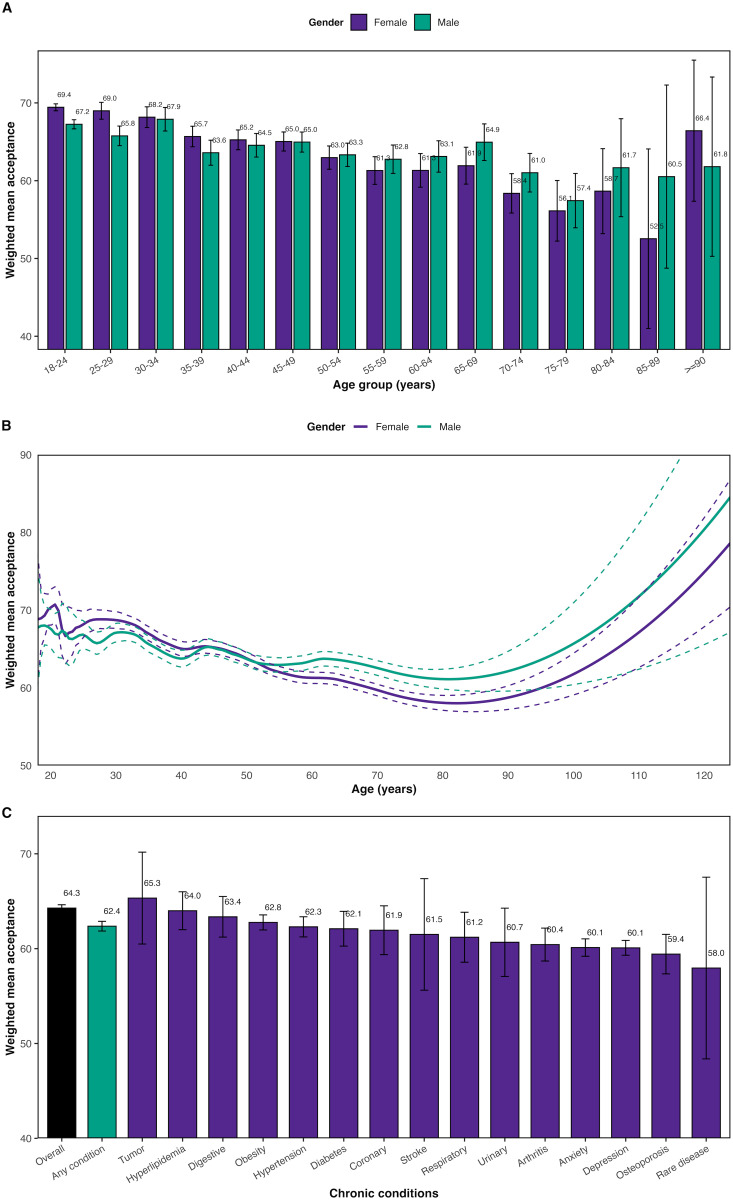
Demographic and clinical stratification of survey-weighted mean acceptance of large language models (LLMs) in healthcare (n = 35,861). **(A)** Weighted mean acceptance by sex and age group. **(B)** Age-related trends in weighted mean acceptance by sex, visualized using LOESS smoothing. **(C)** Weighted mean acceptance among respondents with specific chronic conditions compared with the overall population average. ***Note***: error bars (Panels A, C) and interval lines (Panel B) indicate 95% confidence intervals; LOESS; locally estimated scatterplot smoothing.

Acceptance varied by age (estimates are reliable for participants aged <80 years) but not sex. Acceptance among women peaked at 18–24 years (69.4 [69.0, 70.0]) and acceptance among men at 30–34 years (67.9 [66.4, 69.4]). LOESS curves confirmed minimal sex differences (<4 points) (Fig 1A and 1B and S3 Table).

Respondents with chronic conditions reported lower acceptance than the total sample (62.4 vs. 64.3 overall). Acceptance scores were highest for tumors (65.3), hyperlipidemia (64.0), and digestive disorders (63.4), but lowest for rare diseases (58.0), osteoporosis (59.4), and depression (60.1). Common conditions (obesity, hypertension, diabetes, anxiety) clustered between 60.1 and 62.8 (Fig 1C and S4 Table).

### 3.3 Predictors of acceptance

Of 139 potential predictors, 14 were excluded for multicollinearity (VIF > 3) in their corresponding block models. Among the 127 retained variables, 75 remained significant after FDR correction, showing 92.0% concordance with Elastic-Net selection. Table 2 displays predictors with significant adjusted standardized β-coefficients (β_std) (adjusted p < 0.05); we summarize those with |β_std| ≥ 0.03. Model estimates for non-significant predictors are provided in S5–S11 Tables.

**Table 2:** Significant predictors of acceptance of large language models (LLMs) in healthcare identified by hierarchical weighted linear regression (n = 35,861).

Block/predictor	Standardized β (95% CI)	Adjusted p
**Block 1: sociodemographics**		
Social status	0.19 (0.18, 0.20)	< 0.001
Residence (last 3 months): urban vs. rural	0.05 (0.03, 0.06)	< 0.001
House area	0.02 (0.01, 0.04)	0.007
Number of properties owned	0.02 (0.01, 0.04)	0.003
City tier: new first tier vs. others	0.02 (0.01, 0.03)	0.002
House structure: number of bedrooms	0.02 (0.00, 0.04)	0.028
Per-person income (1–10 levels)*	0.02 (0.00, 0.03)	0.022
Residence duration (1–6 levels)	0.02 (0.00, 0.03)	0.026
Household registration: urban vs. rural*	0.02 (0.00, 0.03)	0.03
Sex orientation: other vs. heterosexual	-0.03 (-0.04, -0.02)	< 0.001
Medical cost difficulty: yes vs. no	-0.04 (-0.05, -0.02)	< 0.001
Religion: yes vs. no	-0.03 (-0.04, -0.01)	< 0.001
Relationship status: widowed vs. single*	-0.01 (-0.02, -0.00)	0.038
Relationship status: married vs. single	-0.03 (-0.05, -0.01)	0.023
Number of siblings	-0.05 (-0.07, -0.04)	< 0.001
Age*	-0.09 (-0.11, -0.08)	< 0.001
**Block 2: life history and adversity**		
Socioeconomic status (youth) (1–7)	0.13 (0.12, 0.15)	< 0.001
ACE: emotional neglect: yes vs. no	0.07 (0.05, 0.08)	< 0.001
Life event: high study/work stress: yes vs. no	0.06 (0.05, 0.08)	< 0.001
ACE: psychological abuse (0–2)	0.06 (0.04, 0.08)	< 0.001
ACE: economic environment (4–20)	0.04 (0.03, 0.06)	< 0.001
Life event: serious injury or illness: yes vs. no	0.02 (0.01, 0.03)	0.001
Life event: legal dispute involvement: yes vs. no	0.02 (0.00, 0.03)	0.013
ACE: older brother died before age 18: yes vs. no	-0.01 (-0.03, -0.00)	0.008
ACE: mother died before age 18: yes vs. no	-0.01 (-0.03, -0.00)	0.015
Life event: difficulty purchasing supplies: yes vs. no	-0.02 (-0.03, -0.01)	0.011
ACE: father died before age 18: yes vs. no	-0.02 (-0.03, -0.01)	0.008
ACE: physical abuse (0–2)	-0.04 (-0.06, -0.02)	< 0.001
ACE: collective violence exposure (0–2)	-0.05 (-0.07, -0.03)	< 0.001
ACE: sexual abuse (0–4)	-0.08 (-0.10, -0.07)	< 0.001
**Block 3: personality and self-efficacy**		
NGSES: self-efficacy (3–15)	0.12 (0.11, 0.13)	< 0.001
NARQ: narcissistic admiration and rivalry (6–36)*	0.03 (0.02, 0.04)	< 0.001
Personality (conscientiousness) (2–10)	0.03 (0.02, 0.04)	< 0.001
Personality (extraversion) (2–10)	-0.02 (-0.03, -0.01)	< 0.001
**Block 4: literacy, empowerment, and social capital**		
Past digital health intervention use: yes vs. no	0.15 (0.14, 0.16)	< 0.001
Family-neighbor relationship (1–7)	0.07 (0.05, 0.08)	< 0.001
eHEALS: eHealth literacy (5–25)	0.06 (0.05, 0.08)	< 0.001
SREBQ: dietary self-regulation (5–25)	0.04 (0.03, 0.06)	< 0.001
PSSS: perceived social support (3–21)	0.04 (0.02, 0.06)	< 0.001
FCS-SF: family communication (4–20)	0.04 (0.02, 0.05)	< 0.001
FHS-SF: family health (10–50)	0.03 (0.01, 0.04)	0.001
HLS-SF: health literacy (0–12)	0.03 (0.01, 0.04)	< 0.001
Social isolation (3–18)	0.02 (0.01, 0.04)	0.005
Antibiotic knowledge: yes vs. no	0.02 (0.01, 0.03)	0.007
Social connection (5–30)*	-0.02 (-0.04, -0.00)	0.019
Social loneliness (2–12)	-0.04 (-0.05, -0.02)	< 0.001
**Block 5: lifestyle factors**		
Sleep hours: 6–7 hours vs. ≤ 6 hours	0.06 (0.04, 0.07)	< 0.001
Sleep hours: 7–8 hours vs. ≤ 6 hours	0.05 (0.03, 0.07)	< 0.001
Exposure to art: yes vs. no	0.03 (0.01, 0.04)	< 0.001
Chronotype: morning vs. evening	-0.02 (-0.03, -0.00)	0.02
Daytime sleepiness: always vs. never/rarely	-0.02 (-0.03, -0.01)	0.007
Extra salt in food: sometimes vs. never/rarely	-0.02 (-0.03, -0.01)	0.003
Sleep difficulty: sometimes vs. never/rarely	-0.02 (-0.04, -0.01)	< 0.001
Daytime sleepiness: sometimes vs. never/rarely	-0.02 (-0.04, -0.01)	0.001
Extra salt in food: often vs. never/rarely	-0.03 (-0.04, -0.01)	< 0.001
Extra salt in food: always vs. never/rarely	-0.03 (-0.04, -0.02)	< 0.001
Smoking habit: yes (conventional cigarettes) vs. no	-0.03 (-0.04, -0.02)	< 0.001
**Block 6: physical health**		
Hepatitis vaccination: yes vs. no	0.03 (0.01, 0.04)	< 0.001
Injury event: sharp‐object injury: yes vs. no	0.02 (0.01, 0.04)	0.001
Injury event: burn or scald: yes vs. no	0.02 (0.01, 0.03)	0.005
Hyperlipidemia diagnosis: yes vs. no	0.02 (0.01, 0.03)	< 0.001
HPV vaccination: yes vs. no	0.02 (0.00, 0.03)	0.015
Injury event: other: yes vs. no	0.02 (0.00, 0.03)	0.007
Injury event: firearm injury: yes vs. no	0.02 (0.00, 0.03)	0.01
Hypertension diagnosis: yes vs. no	0.01 (0.00, 0.02)	0.011
Injury event: suffocation or hanging: yes vs. no	-0.02 (-0.03, -0.00)	0.013
Injury event: motor vehicle accident: yes vs. no	-0.02 (-0.03, -0.01)	0.007
COVID-positive count	-0.02 (-0.03, -0.01)	0.002
**Block 7: mental health**		
CCBI-7: work burnout (7–35)	0.06 (0.04, 0.08)	< 0.001
BSMAS: social media addiction (6–30)	0.05 (0.04, 0.07)	< 0.001
Personal existence (1–7)	0.05 (0.04, 0.07)	< 0.001
SCS: cyberchondria (4–20)	0.04 (0.03, 0.05)	< 0.001
RSS: rest intolerance (8–40)	0.03 (0.01, 0.04)	0.003
MDS5: maladaptive daydreaming (0–100)	-0.04 (-0.05, -0.02)	< 0.001
ASRS-6: ADHD symptoms (0–24)	-0.08 (-0.10, -0.06)	< 0.001

***Note***: Models were adjusted for all predictors retained from previous blocks; variables marked with “*” were not validated by the least absolute shrinkage and selection operator (LASSO); CI, confidence interval; ACE, adverse childhood experiences; NGSES, New General Self-Efficacy Scale; NARQ, Narcissistic Admiration and Rivalry Questionnaire; eHEALS, eHealth Literacy Scale; SREBQ, Self-Regulation of Eating Behavior Questionnaire; PSSS, Perceived Social Support Scale; FCS-SF, Family Communication Scale–Short Form; FHS-SF, Family Health Scale-Short Form; HLS-SF, Health Literacy Scale-Short Form; CCBI-7, 7-item work burnout scale; BSMAS, Bergen Social Media Addiction Scale; SCS, Cyberchondria Scale; RSS, Rest Intolerance Scale; MDS5, 5-item Maladaptive Daydreaming Scale; ASRS-6, 6-item Adult ADHD Self-Report Scale.

#### 3.3.1 Sociodemographics.

Perceived social status was the strongest correlate (β_std = 0.19). Recent urban residence also predicted higher acceptance (β_std = 0.05). Conversely, older age (β_std = -0.09), more siblings (β_std = -0.05), financial strain (β_std = -0.04), being married (β_std = -0.03), religious affiliation (β_std = -0.03), and selecting “other” as sexual orientation compared with being heterosexual (β_std = -0.03) predicted lower acceptance.

#### 3.3.2 Life history and adversity.

High youth SES (β_std = 0.13) was the strongest positive predictor, followed by emotional neglect (β_std = 0.07), psychological abuse (β_std = 0.06), work stress (β_std = 0.06), and advantaged childhood economics status (β_std = 0.04). In contrast, active childhood threats, including sexual abuse (β_std = -0.08), collective violence (β_std = -0.05), and physical abuse (β_std = -0.04), predicted lower acceptance.

#### 3.3.3 Personality and self-efficacy.

Positive predictors included general self-efficacy (β_std = 0.12), conscientiousness (β_std = 0.03), and narcissistic admiration (β_std = 0.03).

#### 3.3.4 Literacy, empowerment, and social capital.

Past digital health experience was the strongest predictor (β_std = 0.15). Other positive predictors included stronger family-neighbor relationships (β_std = 0.07), eHealth literacy (β_std = 0.06), perceived social support (β_std = 0.04), family communication (β_std = 0.04), dietary self-regulation (β_std = 0.04), and general health literacy (β_std = 0.03). Social loneliness predicted lower acceptance (β_std = -0.04).

#### 3.3.5 Lifestyle factors.

Protective behaviors included sleeping 6–8 hours (β_std = 0.05-0.06) and art exposure (β_std = 0.03). Risk factors included high salt intake and current smoking (both β_std = -0.03).

#### 3.3.6 Physical health.

Hepatitis vaccination (β_std = 0.03) showed a positive association. No other physical variables met the pre-specified effect-size threshold.

#### 3.3.7 Mental health.

Acceptance levels were positively correlated with work burnout (β_std = 0.06), social media addiction (β_std = 0.05), sense of personal existence (β_std = 0.05), cyberchondria (β_std = 0.04), and rest intolerance (β_std = 0.03). Negative predictors included severe ADHD symptoms (β_std = -0.08) and maladaptive daydreaming (β_std = -0.04).

### 3.4 Classification-tree analysis of acceptance

**Fig 2:**
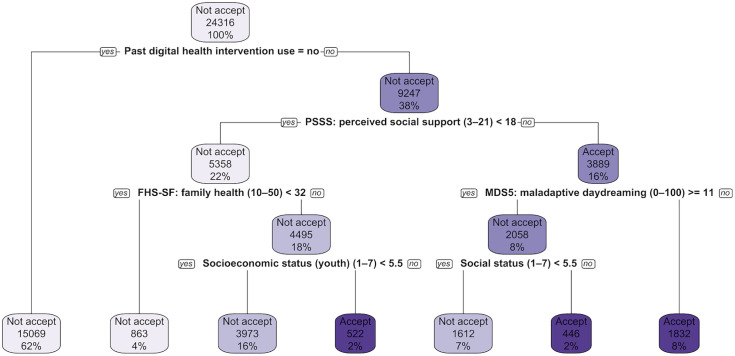
Classification decision tree model of survey-weighted acceptance of large language models (LLMs) in healthcare. The model was fitted to the training set (unweighted n = 23,940; weighted n = 24,316). ***Note***: PSSS; Perceived Social Support Scale (range 3–21); FHS-SF; Family Health Scale-Short Form (range 10–50); MDS5: 5-item Maladaptive Daydreaming Scale (range 0–100).

The CART classification tree (trained on 80% of the sample, n = 24,316) yielded three “acceptor” and four “non-acceptor” profiles (Fig 2). In the test set (n = 5,646), the model achieved modest discrimination (weighted AUC 0.62, 95% CI 0.61-0.64) and 68% accuracy. Specificity was high (0.94) but sensitivity low (0.19) (S2 Fig).

All three acceptor profiles required previous experience with digital health use. The largest group (n = 1,832; 8%) was characterized by high social support (PSSS ≥ 18) and low maladaptive daydreaming (MDS5 < 11). A second profile (n = 522) had low social support but high family health and high childhood SES. The third profile (n = 446) comprised individuals with high social support and high SES despite maladaptive daydreaming.

The dominant non-acceptor pathway, comprising 62% of the sample (n = 15,069), was characterized simply by having no prior experience with digital health use. Among those with prior use, non-acceptance was defined by low social support and poor family health (n = 863), high family health but low youth SES (n = 3,973), or high support and daydreaming but low SES (n = 1,612).

## 4. Discussion

In this national survey of 35,861 adults in China, public acceptance of LLMs in healthcare was moderate overall, with a weighted mean score of 64.3/100. However, this average masked important differences across population subgroups. Acceptance was lower among older adults and among respondents with chronic conditions, and was positively associated with perceived social status, prior digital health use, self-efficacy, social support, and health literacy. By contrast, lower acceptance was observed among those with financial strain, social loneliness, several forms of childhood adversity, and selected neurocognitive and psychological vulnerabilities. These findings suggest that acceptance of LLM-enabled healthcare may be unequally distributed and may be lower in some groups already facing health or social disadvantage.

### 4.1 Patterns of acceptance across regions, age, and chronic conditions

The overall acceptance score in our study is broadly consistent with the cautiously positive but mixed attitudes reported in prior studies of broader AI-enabled healthcare [8,17]. At the same time, we observed considerable regional variation, indicating that public acceptance is unlikely to be uniform across settings. Although some of this variation may reflect differences in regional economic development, digital infrastructure, healthcare access, or prior exposure to digital technologies, our data do not allow firm conclusions about the mechanisms underlying provincial differences. These findings nevertheless suggest that implementation strategies for LLM-enabled healthcare may need to be tailored to local contexts rather than assuming a single national pattern of readiness.

We found little difference in acceptance by sex, which contrasts with some earlier studies suggesting higher acceptance or uptake among men [18]. This discrepancy may reflect differences in study populations, measures, or the distinction between general AI attitudes and acceptance of LLMs in healthcare specifically. In contrast, consistent with broader digital health trends, acceptance declined with age. In China, this reluctance may be compounded by sociocultural and resource barriers: studies of Chinese older adults suggest that digital inclusion is shaped by life-course disadvantage, traditional social roles, limited economic resources, and concerns about cost or device damage, including a culture of thrift that may discourage exploratory technology use [19,20]. Conversely, adults aged ≥90 years exhibited an apparent rebound in acceptance. While this pattern may reflect survivorship bias, with nonagenarians representing a select and potentially more resilient subgroup, the exceptionally wide confidence intervals in this stratum indicate substantial statistical uncertainty. Future research is needed to determine whether this pattern reflects a true cohort-related difference or random sampling variability.

An important finding was that respondents with chronic conditions reported lower acceptance than the overall sample. This pattern does not necessarily mean that people with greater health needs are unwilling to use LLMs in practice, but it does suggest that higher healthcare need does not automatically translate into greater acceptance of these tools. Differences across conditions were also notable. Acceptance was relatively higher in some conditions characterized by ongoing information needs, whereas it was lower in conditions that may involve more complex, sensitive, or relational forms of support, such as depression and rare diseases. These differences should be interpreted cautiously, because our study did not directly assess respondents’ reasons for acceptance or non-acceptance. Nevertheless, they suggest that the perceived usefulness or appropriateness of LLMs may vary across clinical contexts, and that condition-specific implementation may be more acceptable than generic deployment.

### 4.2 Social, developmental, and psychological patterning of acceptance

Our findings suggest a pattern consistent with a potential “Digital Matthew Effect,” in which people with greater social, economic, and digital resources may be more ready to accept LLM-enabled healthcare. At the structural level, unequal digital infrastructure, healthcare access, and technology exposure may shape opportunities to engage with LLM-enabled healthcare. In our study, urban residence may partly reflect these broader structural contexts [21], but it was measured as an individual-level residential characteristic rather than as a direct measure of infrastructure or service availability. At the individual level, psychosocial and economic markers, including higher perceived social status and advantaged childhood socioeconomic position, were associated with higher acceptance, whereas financial strain and religious affiliation were associated with lower acceptance. Among these factors, religion and age have also been reported in prior digital acceptance studies [18,22]. While these findings do not prove that LLM-enabled healthcare will inevitably exacerbate health disparities, they highlight a potential risk: without equity-oriented implementation, early adoption by advantaged groups may reproduce existing inequalities in digital health access and benefit.

Crucially, we identified a novel trauma divergence: survivors of active threats, including sexual abuse, physical abuse, or collective violence, reported significantly lower acceptance, likely reflecting a hyper-vigilance toward systems that could threaten autonomy or safety [23]. In contrast, survivors of emotional neglect showed higher acceptance, seemingly seeking the low-risk, non-judgmental support LLMs could offer [24]. In addition to trauma-related differences, literacy-related disparities were also observed: lower eHealth and general health literacy were associated with lower acceptance of LLMs in healthcare. This suggests that text-heavy or conceptually complex LLM-based systems may disproportionately exclude individuals who have greater difficulty accessing, appraising, and using digital health information. To mitigate these inequalities, distinct strategies are needed: for low-literacy populations, developers should prioritize voice-first interfaces; for trauma survivors, regaining trust requires privacy-first architectures with clear user controls to ensure the system is perceived as safe rather than surveillance-oriented.

Psychological profiling revealed that acceptance was associated with two competing motives: cognitive agency and distress-driven reliance. High acceptance was associated with action-oriented traits (self-efficacy, conscientiousness, rest intolerance, and narcissistic admiration), suggesting that LLM-enabled healthcare may be more acceptable to those eager to actively manage their health. Our observation that self-efficacy was positively associated with acceptance of LLMs in healthcare aligns with mHealth research [25], while links to conscientiousness and rest intolerance imply that LLM-based tools may be perceived as efficient channels for health information processing [26]. Conversely, acceptance was also associated with indicators of psychological strain (work stress, cyberchondria, and social media addiction), which may reflect greater interest in LLM-enabled health information support for reassurance among digitally engaged users. However, neurocognitive vulnerabilities were associated with lower acceptance: severe ADHD symptoms and maladaptive daydreaming strongly predicted lower acceptance. This pattern is consistent with the possibility that text-heavy, high-friction LLM-based tools may be less appealing to users with attentional or cognitive difficulties [27], supporting future interface designs that reduce cognitive burden for neurodivergent users.

Finally, our results highlighted a “connectivity and literacy” divide. People with higher eHealth literacy and strong social support networks were more likely to accept LLMs, consistent with evidence characterizing literacy [28] and social capital [29] as prerequisites for digital uptake. Crucially, those who felt socially isolated or lonely had lower acceptance, aligning with the link between social isolation and digital exclusion [30]. Thus, acceptance appeared higher among the digitally competent and socially connected. Health behaviors reinforced this gradient: cultural engagement (e.g., art exposure) correlated with acceptance, likely reflecting broader traits of intellectual curiosity [31]. Conversely, risk behaviors (smoking, high salt intake), were associated with lower acceptance, likely reflecting a broader profile often associated with lower SES [32]. To engage populations with specific risk profiles, implementation strategies must shift from merely lowering access barriers to actively enhancing the technology’s perceived utility. Future LLM-enabled healthcare tools should integrate real-time, actionable support, such as conversational dietary trackers or personalized, non-judgmental nudges for behavioral change, to overcome these groups’ distinct motivational barriers to acceptance.

### 4.3 Interpretation of the classification tree

The classification tree provides an interpretable summary of how selected factors combine to differentiate respondents with lower versus higher acceptance. The most notable finding was that lack of prior digital health use defined the largest non-acceptor group, accounting for 62% of the sample. Among respondents with prior digital experience, lower social support, poorer family health, lower childhood socioeconomic status, and maladaptive daydreaming further distinguished groups with lower acceptance. These patterns are broadly consistent with the regression analyses and reinforce the importance of digital exposure and social resources.

However, the tree should be interpreted carefully. Its discrimination was modest, with a weighted AUC of 0.62 and 68% accuracy, and its low sensitivity indicates limited predictive utility. Because acceptance was the predicted outcome, the low sensitivity indicates that the model captured only a small proportion of acceptors, whereas the high specificity suggests that it was better at identifying non-acceptors. Thus, the apparent accuracy should not be overinterpreted, as it may partly reflect the predominance of non-acceptance profiles. Accordingly, it should not be considered a strong predictive model of individual acceptance. Rather, its main value lies in providing an accessible, descriptive framework for identifying combinations of characteristics associated with acceptance and non-acceptance. From an implementation perspective, this may help generate hypotheses about population subgroups that may require additional engagement, communication, or support, but the tree should not be used alone to determine individual readiness for LLM-enabled healthcare or to guide resource allocation.

### 4.4 Implications for equitable implementation

Our findings have implications for how LLM-enabled healthcare are introduced into healthcare systems. First, moderate average acceptance should not be taken to imply broad readiness across all subgroups. The observed gradients by age, chronic illness, social position, digital experience, and psychosocial context suggest that uptake may be uneven if implementation relies on passive diffusion alone. Therefore, LLM-enabled healthcare should not be deployed only as a universal digital service and assumed to diffuse equally across the population.

Second, implementation strategies may need to go beyond universal availability and consider targeted approaches for groups less likely to engage. These could include clearer explanations of what LLM-based tools can and cannot do, interfaces that reduce literacy or cognitive burden, and deployment models that preserve human support rather than replacing it. At the communication level, public-facing materials should explain LLM functions, limitations, privacy protections, and when users should seek professional care, using plain language and concrete examples. At the access level, assisted onboarding could be provided through community health centers, primary care clinics, older adult services, or family/caregiver-supported settings, especially for older adults, people with chronic conditions, and those without prior digital health use. At the interface level, tools should include voice-first options, simplified navigation, low-literacy explanations, and clear escalation pathways to human professionals. These findings are also relevant to the implementation of LLM-enabled healthcare in low-resource settings, where barriers may include limited digital infrastructure, low eHealth literacy, affordability concerns, and weaker access to professional support. In such settings, LLM-enabled tools may be more equitable if delivered through low-bandwidth platforms, shared community access points, primary care or community-health-worker support, and voice-based or local-language interfaces rather than standalone text-heavy applications.

Third, evaluation of LLM-enabled services should include equity-sensitive outcomes, including who adopts these tools, who does not, and whether their use narrows or widens existing disparities in access and benefit. Such monitoring should be stratified by age, socioeconomic position, chronic disease status, literacy, social support, and prior digital health use. In addition to uptake, evaluations should track discontinuation, unmet support needs, safety concerns, user trust, and whether clinical or informational benefits are equitably distributed. These steps would help ensure that LLM-enabled healthcare improves access rather than reinforcing existing digital and health inequities.

### 4.5 Strengths and limitations

This study has several strengths. It uses, to our knowledge, the largest population-based dataset on public acceptance of LLMs in healthcare in China to date, with broad geographic coverage and a large set of candidate predictors spanning multiple domains. The use of survey weighting, hierarchical regression, Elastic-Net validation, and classification-tree analysis allowed us to examine both independent associations and interpretable profiles of non-acceptance.

Several limitations should also be considered. First, the cross-sectional design precludes causal inference, and the observed associations may reflect bidirectional or unmeasured relationships. Second, acceptance was measured using a single self-report item after a standardized description of LLMs. Therefore, it should be interpreted as hypothetical stated acceptance or readiness, rather than as a validated multidimensional measure of trust, perceived usefulness, intention to use, or real-world adoption. Prior familiarity with LLMs may also have influenced responses. Future studies should use multidimensional scales, behavioral data, and qualitative methods to validate and extend these findings. Third, the electronic format of the questionnaire may have underrepresented individuals with the greatest levels of digital exclusion or literacy difficulty, which is particularly relevant given the focus of this study. Fourth, all variables were self-reported and therefore subject to recall bias and social desirability bias, especially for sensitive experiences such as childhood adversity and mental health symptoms. Finally, the classification tree had modest discrimination and should be viewed as a descriptive tool rather than a precise predictive model.

## 5. Conclusions

In this national survey of adults in China, acceptance of LLMs in healthcare was moderate overall but varied substantially across population subgroups. Lower acceptance was observed among older adults, respondents with chronic conditions, and those with fewer social, developmental, and digital resources. Higher acceptance was associated with prior digital health use, stronger social support, higher perceived social status, and greater self-efficacy and health literacy. These findings suggest that the diffusion of LLM-enabled healthcare may not be equitable by default. As LLMs become more integrated into healthcare, implementation should be assessed not only in terms of overall uptake, but also in terms of who is able and willing to engage. Strategies to support equitable adoption may include accessible communication, reduced cognitive and literacy burden, preservation of human support, and targeted outreach to groups less likely to accept these tools. Future research should examine whether differences in acceptance translate into differences in actual use, quality of care, and health outcomes.

## Supporting information

S1 Fig Flow diagram of data screening and participant selection. (DOCX)

S2 Fig ROC curve for the CART model in the test set, including shaded area under the curve and annotated AUC (95% bootstrap CI). (DOCX)

S1 Table Definitions, measurement ranges, and reliability metrics for all predictor variables across seven blocks. (DOCX)

S2 Table Unweighted and weighted sample sizes and weighted acceptance of large language model healthcare by province (n = 35,861). (DOCX)

S3 Table Unweighted and weighted counts and weighted mean acceptance of large language model healthcare by age group and gender (n = 35,861). (DOCX)

S4 Table Unweighted and weighted sample sizes and weighted mean acceptance of large language model healthcare by health condition (n = 35,861). (DOCX)

S5 Table Block 1: hierarchical weighted linear regression of sociodemographics on acceptance of large language model in healthcare (n = 35,861). (DOCX)

S6 Table Block 2: hierarchical weighted linear regression of life adversity and adverse childhood experience predictors on acceptance of large language model in healthcare (n = 35,861). (DOCX)

S7 Table Block 3: hierarchical weighted linear regression of personality predictors (Block 3) on acceptance of large language model in healthcare (n = 35,861). (DOCX)

S8 Table Block 4: hierarchical weighted linear regression of literacy and empowerment predictors on acceptance of large language model in healthcare (n = 35,861). (DOCX)

S9 Table Block 5: hierarchical weighted linear regression of lifestyle factors on acceptance of large language model in healthcare (n = 35,861). (DOCX)

S10 Table Block 6: hierarchical weighted linear regression of physical health predictors on acceptance of large language model in healthcare (n = 35,861). (DOCX)

S11 Table Block 7: hierarchical weighted linear regression of mental-health predictors on acceptance of large language model in healthcare (n = 35,861). (DOCX)
