# Supplementary figures and images for: Health equity and public acceptance of large language models in healthcare in China: A national population-based survey
Source: PLOS Digit Health. 2026 Jul 30;5(7):e0001555. doi: 10.1371/journal.pdig.0001555 (PMC13422829; doi:10.1371/journal.pdig.0001555)

**S1 Figure.** Flow diagram of data screening and participant selection.


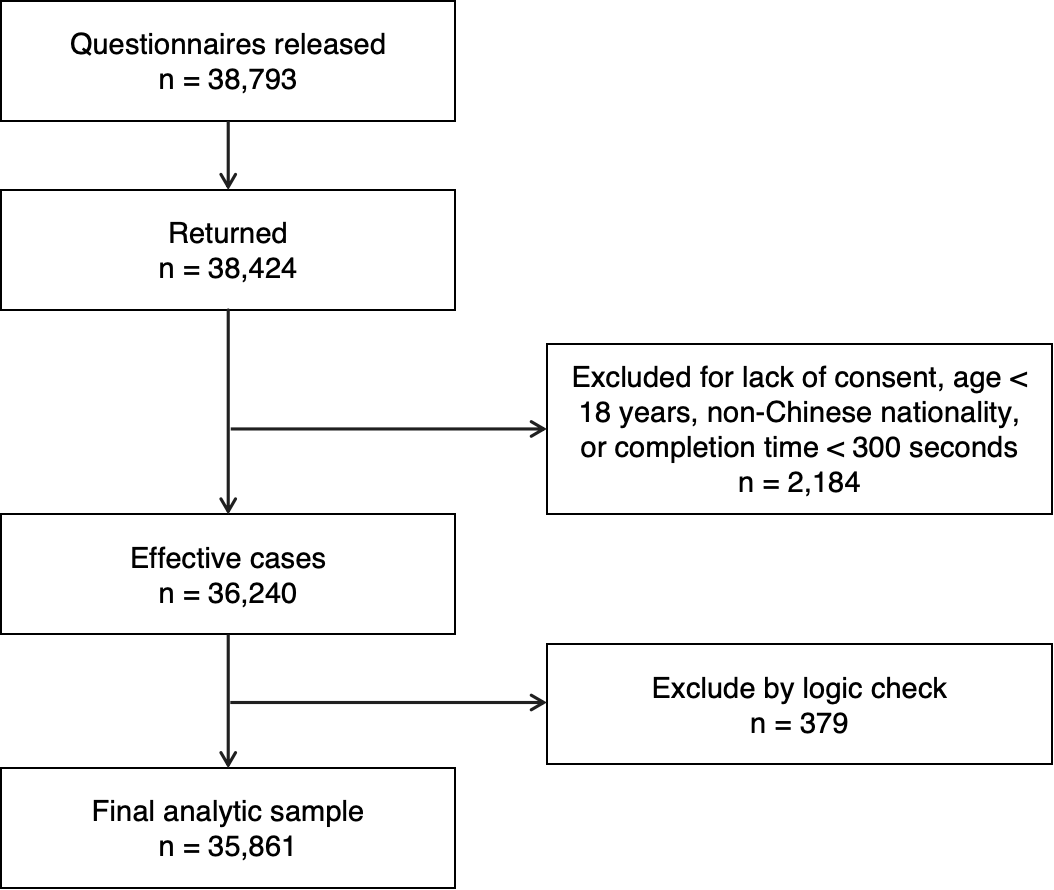

Supplement: S1 Fig — (DOCX) [file pdig.0001555.s001.docx]
