# Supplementary material for: Health equity and public acceptance of large language models in healthcare in China: A national population-based survey
Source: PLOS Digit Health. 2026 Jul 30;5(7):e0001555. doi: 10.1371/journal.pdig.0001555 (PMC13422829; doi:10.1371/journal.pdig.0001555)

**S2 Figure.** ROC curve for the CART model in the test set, including shaded area under the curve and annotated AUC (95% bootstrap CI).


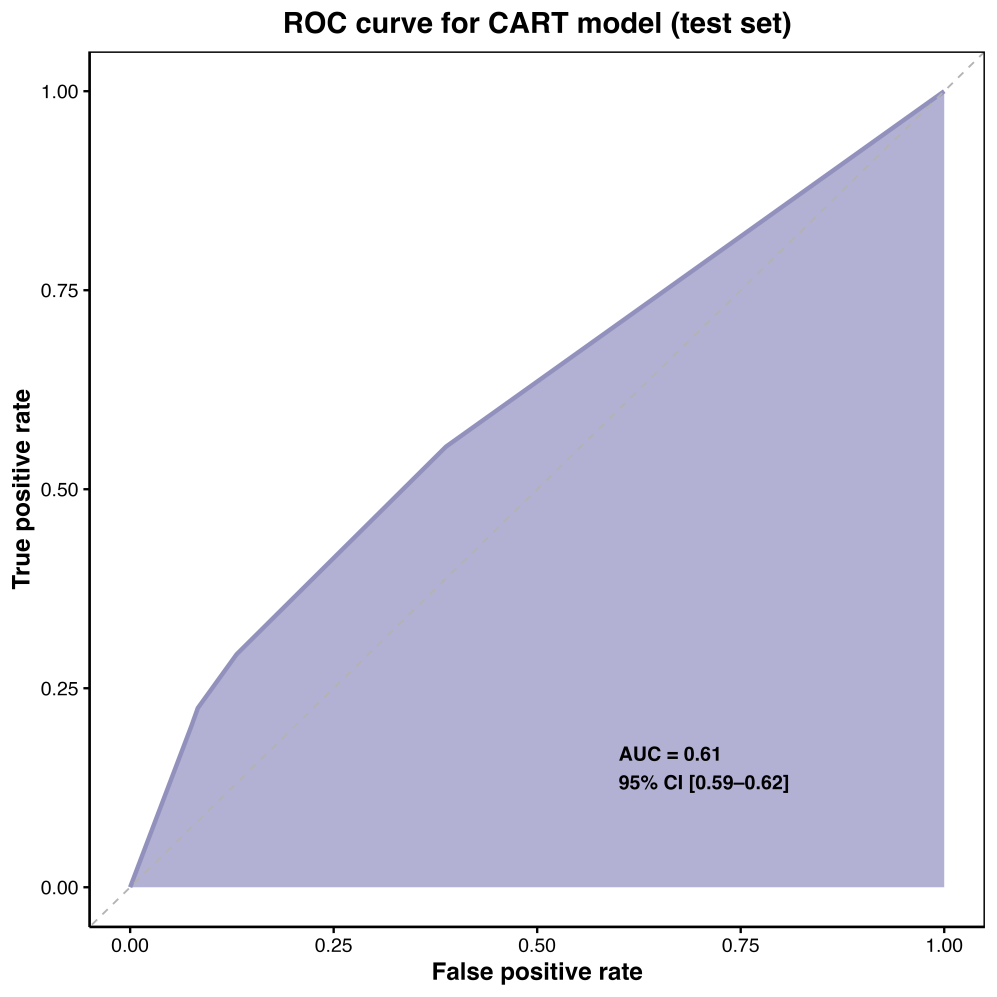

Supplement: S2 Fig — (DOCX) [file pdig.0001555.s002.docx]
