# Supplementary material for: Health equity and public acceptance of large language models in healthcare in China: A national population-based survey
Source: PLOS Digit Health. 2026 Jul 30;5(7):e0001555. doi: 10.1371/journal.pdig.0001555 (PMC13422829; doi:10.1371/journal.pdig.0001555)
Supplement: S2 Table — (DOCX) [file pdig.0001555.s004.docx]

**S2 Table.** Unweighted and weighted sample sizes and weighted acceptance of large language model healthcare by province (n=35,861).

| Province | Unweighted n | Weighted n | Weighted acceptance (95% CI) |
| --- | --- | --- | --- |
| Jilin | 122 | 65 | 72·25 (65·51, 79·00) |
| Liaoning | 430 | 335 | 71·94 (68·59, 75·28) |
| Jiangsu | 973 | 906 | 69·38 (67·31, 71·45) |
| Tianjin | 395 | 406 | 67·67 (64·40, 70·94) |
| Shanghai | 886 | 1225 | 67·24 (65·27, 69·22) |
| Guangdong | 938 | 1168 | 67·23 (65·02, 69·45) |
| Hebei | 1040 | 888 | 66·34 (64·30, 68·38) |
| Shandong | 4677 | 4689 | 65·98 (65·02, 66·94) |
| Anhui | 4641 | 6306 | 65·65 (64·83, 66·48) |
| Henan | 1906 | 2094 | 65·27 (63·84, 66·71) |
| Fujian | 1068 | 1341 | 64·99 (63·05, 66·93) |
| Hainan | 278 | 246 | 64·94 (61·67, 68·21) |
| Zhejiang | 335 | 378 | 63·89 (60·61, 67·16) |
| Xinjiang | 226 | 231 | 63·76 (59·54, 67·98) |
| Chongqing | 655 | 729 | 63·73 (61·43, 66·03) |
| Sichuan | 2509 | 2958 | 63·59 (62·44, 64·74) |
| Ningxia | 794 | 939 | 63·59 (61·41, 65·77) |
| Heilongjiang | 1521 | 1022 | 63·56 (61·79, 65·33) |
| Yunnan | 1154 | 762 | 63·11 (60·52, 65·71) |
| Beijing | 941 | 1138 | 63·04 (60·96, 65·12) |
| Guangxi | 416 | 358 | 62·71 (59·43, 65·98) |
| Hunan | 912 | 760 | 61·60 (59·10, 64·11) |
| Hubei | 1017 | 877 | 61·58 (59·37, 63·79) |
| Shaanxi | 326 | 172 | 61·14 (56·61, 65·67) |
| Jiangxi | 3881 | 2881 | 60·79 (59·48, 62·09) |
| Qinghai | 393 | 405 | 59·94 (56·26, 63·63) |
| Gansu | 441 | 439 | 59·66 (56·25, 63·07) |
| Guizhou | 563 | 451 | 58·20 (55·53, 60·87) |
| Shanxi | 607 | 725 | 57·31 (54·88, 59·74) |
| Tibet | 1317 | 440 | 57·09 (54·84, 59·35) |
| Inner Mongolia | 54 | 42 | 55·50 (43·06, 67·94) |
| Hong Kong | 12 | 12 | 49·49 (29·25, 69·74) |
| Macau | 12 | 22 | 43·07 (20·91, 65·24) |
