# Supplementary material for: Health equity and public acceptance of large language models in healthcare in China: A national population-based survey
Source: PLOS Digit Health. 2026 Jul 30;5(7):e0001555. doi: 10.1371/journal.pdig.0001555 (PMC13422829; doi:10.1371/journal.pdig.0001555)
Supplement: S3 Table — (DOCX) [file pdig.0001555.s005.docx]

**S3 Table.** Unweighted and weighted counts and weighted mean acceptance of large language model healthcare by age group and gender (n=35,861).

| **Age group (years)** | **Female** | | | **Male** | | |
| --- | --- | --- | --- | --- | --- | --- |
|  | **n** | **Weighted n** | **Weighted acceptance (95% CI)** | **n** | **Weighted n** | **Weighted acceptance**  **(95% CI)** |
| 18-24 | 9302 | 1166 | 69·43 (68·99, 69·88) | 6103 | 1340 | 67·25 (66·66, 67·83) |
| 25-29 | 1832 | 1444 | 68·98 (67·90, 70·07) | 1393 | 1606 | 65·76 (64·50, 67·02) |
| 30-34 | 1263 | 1991 | 68·16 (66·83, 69·49) | 958 | 2120 | 67·89 (66·38, 69·40) |
| 35-39 | 1368 | 1588 | 65·67 (64·36, 66·99) | 991 | 1686 | 63·59 (61·98, 65·20) |
| 40-44 | 1325 | 1497 | 65·25 (63·98, 66·52) | 1037 | 1575 | 64·55 (63·03, 66·06) |
| 45-49 | 1595 | 1851 | 65·03 (63·81, 66·26) | 1409 | 1923 | 64·96 (63·67, 66·25) |
| 50-54 | 1105 | 1984 | 62·96 (61·47, 64·46) | 1070 | 2019 | 63·32 (61·83, 64·82) |
| 55-59 | 770 | 1671 | 61·30 (59·52, 63·08) | 751 | 1679 | 62·76 (60·93, 64·58) |
| 60-64 | 540 | 1206 | 61·32 (59·16, 63·48) | 554 | 1218 | 63·11 (61·09, 65·13) |
| 65-69 | 446 | 1244 | 61·93 (59·56, 64·30) | 453 | 1200 | 64·94 (62·59, 67·30) |
| 70-74 | 469 | 840 | 58·37 (55·84, 60·90) | 456 | 798 | 61·02 (58·54, 63·50) |
| 75-79 | 198 | 545 | 56·13 (52·23, 60·02) | 222 | 487 | 57·44 (53·95, 60·92) |
| 80-84 | 95 | 371 | 58·65 (53·19, 64·11) | 75 | 302 | 61·67 (55·37, 67·96) |
| 85-89 | 24 | 211 | 52·54 (41·01, 64·08) | 23 | 146 | 60·52 (48·75, 72·29) |
| ≥90 | 19 | 96 | 66·42 (57·35, 75·49) | 15 | 55 | 61·80 (50·28, 73·32) |

***Note*:** CI: confidence interval.
