# Supplementary material for: Health equity and public acceptance of large language models in healthcare in China: A national population-based survey
Source: PLOS Digit Health. 2026 Jul 30;5(7):e0001555. doi: 10.1371/journal.pdig.0001555 (PMC13422829; doi:10.1371/journal.pdig.0001555)
Supplement: S4 Table — (DOCX) [file pdig.0001555.s006.docx]

**S4 Table.** Unweighted and weighted sample sizes and weighted mean acceptance of large language model healthcare by health condition (n=35,861).

| **Condition** | **n** | **Weighted n** | **Weighted acceptance (95% CI)** |
| --- | --- | --- | --- |
| Overall | 35861 | 35861 | 64·27 (63·92, 64·63) |
| Any condition | 16729 | 19397 | 62·37 (61·85, 62·88) |
| Hypertension | 2816 | 5687 | 62·30 (61·24, 63·36) |
| Diabetes | 993 | 1990 | 62·09 (60·26, 63·93) |
| Hyperlipidemia | 791 | 1509 | 64·00 (62·00, 65·99) |
| Coronary | 453 | 938 | 61·94 (59·37, 64·52) |
| Stroke | 110 | 202 | 61·50 (55·62, 67·38) |
| Respiratory | 511 | 770 | 61·20 (58·57, 63·84) |
| Urinary | 253 | 403 | 60·67 (57·07, 64·27) |
| Digestive | 814 | 1094 | 63·36 (61·21, 65·50) |
| Osteoporosis | 798 | 1591 | 59·42 (57·34, 61·50) |
| Arthritis | 1060 | 1944 | 60·43 (58·70, 62·16) |
| Tumor | 196 | 292 | 65·33 (60·48, 70·17) |
| Obesity | 6156 | 7585 | 62·76 (61·97, 63·56) |
| Rare disease | 69 | 71 | 57·95 (48·38, 67·53) |
| Anxiety | 6129 | 5160 | 60·11 (59·20, 61·03) |
| Depression | 8192 | 6891 | 60·08 (59·30, 60·86) |

***Note***: CI: confidence interval.
