# Supplementary material for: Health equity and public acceptance of large language models in healthcare in China: A national population-based survey
Source: PLOS Digit Health. 2026 Jul 30;5(7):e0001555. doi: 10.1371/journal.pdig.0001555 (PMC13422829; doi:10.1371/journal.pdig.0001555)
Supplement: S5 Table — (DOCX) [file pdig.0001555.s007.docx]

**S5 Table.** Block 1: hierarchical weighted linear regression of sociodemographics on acceptance of large language model in healthcare (n=35,861).

| **Predictor** | **Standardized β (95% CI)** | **p** | **Adjusted p** |
| --- | --- | --- | --- |
| Age | -0·09 (-0·11, -0·08) | < 0·001 | < 0·001 |
| City tier: first tier vs· others | 0·00 (-0·01, 0·01) | 0·988 | 0·988 |
| City tier: new first tier vs· others | 0·02 (0·01, 0·03) | 0·001 | 0·002 |
| Handedness: right hand vs· left hand | 0·01 (0·00, 0·02) | 0·044 | 0·069 |
| House area | 0·02 (0·01, 0·04) | 0·003 | 0·007 |
| House structure: number of bedrooms | 0·02 (0·00, 0·04) | 0·014 | 0·028 |
| Household registration: urban vs· rural | 0·02 (0·00, 0·03) | 0·016 | 0·030 |
| Lived alone in past 3 months: yes vs· no | -0·01 (-0·02, 0·01) | 0·271 | 0·361 |
| Medical cost difficulty: yes vs· no | -0·04 (-0·05, -0·02) | < 0·001 | < 0·001 |
| Medicare enrollment: living area vs· Hukou location | 0·01 (0·00, 0·03) | 0·039 | 0·064 |
| Medicare enrollment: others vs· Hukou location | -0·02 (-0·03, 0·00) | 0·054 | 0·079 |
| No debt: yes vs· no | 0·00 (-0·01, 0·02) | 0·549 | 0·570 |
| Number of properties owned | 0·02 (0·01, 0·04) | 0·001 | 0·003 |
| Number of siblings | -0·05 (-0·07, -0·04) | < 0·001 | < 0·001 |
| Per-person income | 0·02 (0·00, 0·03) | 0·009 | 0·022 |
| Race: others vs· Han | -0·01 (-0·02, 0·01) | 0·340 | 0·433 |
| Relationship status: divorced vs· single | 0·00 (-0·01, 0·01) | 0·534 | 0·570 |
| Relationship status: in relationship vs· single | -0·01 (-0·03, 0·01) | 0·445 | 0·519 |
| Relationship status: married vs· single | -0·03 (-0·05, -0·01) | 0·010 | 0·023 |
| Relationship status: widowed vs· single | -0·01 (-0·02, -0·00) | 0·022 | 0·038 |
| Religion: yes vs· no | -0·03 (-0·04, -0·01) | < 0·001 | < 0·001 |
| Residence (last 3 months): urban vs· rural | 0·05 (0·03, 0·06) | < 0·001 | < 0·001 |
| Residence duration | 0·02 (0·00, 0·03) | 0·012 | 0·026 |
| Sex orientation: asexual vs· heterosexual | -0·00 (-0·02, 0·01) | 0·442 | 0·519 |
| Sex orientation: bisexual vs· heterosexual | -0·01 (-0·03, 0·00) | 0·092 | 0·128 |
| Sex orientation: homosexual vs· heterosexual | 0·00 (-0·01, 0·02) | 0·538 | 0·570 |
| Sex orientation: other vs· heterosexual | -0·03 (-0·04, -0·02) | < 0·001 | < 0·001 |
| Social status | 0·19 (0·18, 0·20) | < 0·001 | < 0·001 |

***Note***: CI, confidence interval; City tier (first tier, new first tier): Chinese city classification by urbanization and economic development; Hukou: household registration location.
