# Supplementary material for: Health equity and public acceptance of large language models in healthcare in China: A national population-based survey
Source: PLOS Digit Health. 2026 Jul 30;5(7):e0001555. doi: 10.1371/journal.pdig.0001555 (PMC13422829; doi:10.1371/journal.pdig.0001555)
Supplement: S6 Table — (DOCX) [file pdig.0001555.s008.docx]

**S6 Table.** Block 2: hierarchical weighted linear regression of life adversity and adverse childhood experience predictors on acceptance of large language model in healthcare (n=35,861).

| **Predictor** | **Standardized β (95% CI)** | **p** | **Adjusted p** |
| --- | --- | --- | --- |
| ACE: collective violence exposure (0–2) | -0·05 (-0·07, -0·03) | < 0·001 | < 0·001 |
| ACE: community violence exposure (0–2) | 0·01 (-0·00, 0·02) | 0·186 | 0·258 |
| ACE: economic environment (4–20) | 0·04 (0·03, 0·06) | < 0·001 | < 0·001 |
| ACE: emotional neglect: yes vs· no | 0·07 (0·05, 0·08) | < 0·001 | < 0·001 |
| ACE: father died before age 18: yes vs· no | -0·02 (-0·03, -0·01) | 0·003 | 0·008 |
| ACE: mother died before age 18: yes vs· no | -0·01 (-0·03, -0·00) | 0·007 | 0·015 |
| ACE: older brother died before age 18: yes vs· no | -0·01 (-0·03, -0·00) | 0·003 | 0·008 |
| ACE: older sister died before age 18: yes vs· no | 0·00 (-0·01, 0·01) | 0·695 | 0·725 |
| ACE: physical abuse (0–2) | -0·04 (-0·06, -0·02) | < 0·001 | < 0·001 |
| ACE: psychological abuse (0–2) | 0·06 (0·04, 0·08) | < 0·001 | < 0·001 |
| ACE: sexual abuse (0–4) | -0·08 (-0·10, -0·07) | < 0·001 | < 0·001 |
| ACE: witnessed maternal violence (0–4) | -0·02 (-0·04, 0·00) | 0·071 | 0·117 |
| ACE: younger brother died before age 18: yes vs· no | 0·01 (-0·00, 0·02) | 0·292 | 0·385 |
| ACE: younger sister died before age 18: yes vs· no | -0·01 (-0·02, 0·00) | 0·134 | 0·196 |
| Life event: accident or natural disaster: yes vs· no | 0·01 (-0·00, 0·02) | 0·224 | 0·302 |
| Life event: difficulty purchasing supplies: yes vs· no | -0·02 (-0·03, -0·01) | 0·004 | 0·011 |
| Life event: family conflict: yes vs· no | 0·01 (-0·00, 0·03) | 0·059 | 0·103 |
| Life event: family economic hardship: yes vs· no | 0·01 (-0·01, 0·02) | 0·335 | 0·421 |
| Life event: high study/work stress: yes vs· no | 0·06 (0·05, 0·08) | < 0·001 | < 0·001 |
| Life event: immediate family death: yes vs· no | -0·01 (-0·02, -0·00) | 0·047 | 0·087 |
| Life event: legal dispute involvement: yes vs· no | 0·02 (0·00, 0·03) | 0·006 | 0·013 |
| Life event: major change in routine: yes vs· no | 0·01 (-0·01, 0·02) | 0·322 | 0·415 |
| Life event: other: yes vs· no | -0·01 (-0·02, 0·00) | 0·052 | 0·093 |
| Life event: serious injury or illness: yes vs· no | 0·02 (0·01, 0·03) | < 0·001 | 0·001 |
| Life event: theft or property loss: yes vs· no | -0·01 (-0·02, 0·00) | 0·119 | 0·178 |
| Life event: unable to reunite with family: yes vs· no | -0·00 (-0·02, 0·01) | 0·435 | 0·534 |
| Life event: unemployment: yes vs· no | 0·00 (-0·01, 0·01) | 0·614 | 0·664 |
| Socioeconomic status (youth) (1–7) | 0·13 (0·12, 0·15) | < 0·001 | < 0·001 |

***Note***: CI, confidence interval; ACE, adverse childhood experiences.
