# Supplementary material for: Health equity and public acceptance of large language models in healthcare in China: A national population-based survey
Source: PLOS Digit Health. 2026 Jul 30;5(7):e0001555. doi: 10.1371/journal.pdig.0001555 (PMC13422829; doi:10.1371/journal.pdig.0001555)
Supplement: S7 Table — (DOCX) [file pdig.0001555.s009.docx]

**S7 Table.** Block 3: hierarchical weighted linear regression of personality predictors (Block 3) on acceptance of large language model in healthcare (n = 35,861).

| **Predictor** | **Standardized β (95% CI)** | **p** | **Adjusted p** |
| --- | --- | --- | --- |
| Personality (extraversion) (2–10) | -0·02 (-0·03, -0·01) | < 0·001 | < 0·001 |
| NARQ: narcissistic admiration & rivalry (6–36) | 0·03 (0·02, 0·04) | < 0·001 | < 0·001 |
| NGSES: self-efficacy (3–15) | 0·12 (0·11, 0·13) | < 0·001 | < 0·001 |
| Personality (agreeableness) (2–10) | -0·01 (-0·02, 0·00) | 0·269 | 0·332 |
| Personality (conscientiousness) (2–10) | 0·03 (0·02, 0·04) | < 0·001 | < 0·001 |
| Personality (neuroticism) (2–10) | 0·00 (-0·01, 0·01) | 0·848 | 0·866 |
| Personality (openness) (2–10) | -0·01 (-0·02, 0·00) | 0·062 | 0·091 |

***Note***: CI, confidence interval; NARQ, Narcissistic Admiration and Rivalry Questionnaire; eHEALS, eHealth Literacy Scale.
