# Supplementary material for: Health equity and public acceptance of large language models in healthcare in China: A national population-based survey
Source: PLOS Digit Health. 2026 Jul 30;5(7):e0001555. doi: 10.1371/journal.pdig.0001555 (PMC13422829; doi:10.1371/journal.pdig.0001555)
Supplement: S8 Table — (DOCX) [file pdig.0001555.s010.docx]

**S8 Table.** Block 4: hierarchical weighted linear regression of literacy and empowerment predictors on acceptance of large language model in healthcare (n=35,861).

| **Predictor** | **Standardized β (95% CI)** | **p** | **Adjusted p** |
| --- | --- | --- | --- |
| Family-neighbor relationship (1–7) | 0·07 (0·05, 0·08) | < 0·001 | < 0·001 |
| Antibiotic knowledge: yes vs· no | 0·02 (0·01, 0·03) | 0·003 | 0·007 |
| eHEALS: ehealth literacy (5–25) | 0·06 (0·05, 0·08) | < 0·001 | < 0·001 |
| FCS-SF: family communication (4–20) | 0·04 (0·02, 0·05) | < 0·001 | < 0·001 |
| FHS-SF: family health (10–50) | 0·03 (0·01, 0·04) | < 0·001 | 0·001 |
| Have someone to trust: yes vs· no | -0·01 (-0·02, 0·00) | 0·137 | 0·207 |
| HLS-SF: health literacy (0–12) | 0·03 (0·01, 0·04) | < 0·001 | < 0·001 |
| Implicit health beliefs (6–36) | -0·00 (-0·01, 0·01) | 0·715 | 0·769 |
| Past digital health intervention use: yes vs· no | 0·15 (0·14, 0·16) | < 0·001 | < 0·001 |
| PSSS: perceived social support (3–21) | 0·04 (0·02, 0·06) | < 0·001 | < 0·001 |
| Received help when needed: yes vs· no | -0·00 (-0·01, 0·01) | 0·743 | 0·769 |
| Social connection (5–30) | -0·02 (-0·04, -0·00) | 0·01 | 0·019 |
| Social isolation (3–18) | 0·02 (0·01, 0·04) | 0·002 | 0·005 |
| Social loneliness (2–12) | -0·04 (-0·05, -0·02) | < 0·001 | < 0·001 |
| SREBQ: dietary self-regulation (5–25) | 0·04 (0·03, 0·06) | < 0·001 | < 0·001 |

***Note***: CI, confidence interval; eHEALS, eHealth Literacy Scale; SREBQ, Self-Regulation of Eating Behavior Questionnaire.
