# Supplementary material for: Health equity and public acceptance of large language models in healthcare in China: A national population-based survey
Source: PLOS Digit Health. 2026 Jul 30;5(7):e0001555. doi: 10.1371/journal.pdig.0001555 (PMC13422829; doi:10.1371/journal.pdig.0001555)
Supplement: S9 Table — (DOCX) [file pdig.0001555.s011.docx]

**S9 Table.** Block 5: hierarchical weighted linear regression of lifestyle factors on acceptance of large language model in healthcare (n=35,861).

| **Predictor** | **Standardized β (95% CI)** | **p** | **Adjusted p** |
| --- | --- | --- | --- |
| Alcohol use: current drinker (including occasional) vs· never drink | 0·00 (-0·01, 0·01) | 0·786 | 0·829 |
| Alcohol use: former drinker vs· never drink | 0·00 (-0·01, 0·01) | 0·865 | 0·897 |
| Alcohol use: new drinker vs· never drink | 0·00 (-0·01, 0·02) | 0·789 | 0·829 |
| Chronotype: morning vs· evening | -0·02 (-0·03, -0·00) | 0·01 | 0·02 |
| Daytime sleepiness: always vs· never/rarely | -0·02 (-0·03, -0·01) | 0·003 | 0·007 |
| Daytime sleepiness: often vs· never/rarely | -0·01 (-0·02, 0·00) | 0·127 | 0·181 |
| Daytime sleepiness: sometimes vs· never/rarely | -0·02 (-0·04, -0·01) | < 0·001 | 0·001 |
| Exposure to art: yes vs· no | 0·03 (0·01, 0·04) | < 0·001 | < 0·001 |
| Exposure to dance: yes vs· no | 0·01 (-0·01, 0·02) | 0·388 | 0·473 |
| Exposure to music: yes vs· no | -0·01 (-0·03, 0·00) | 0·078 | 0·118 |
| Exposure to other arts: yes vs· no | 0·00 (-0·01, 0·02) | 0·59 | 0·644 |
| Extra salt in food: always vs· never/rarely | -0·03 (-0·04, -0·02) | < 0·001 | < 0·001 |
| Extra salt in food: often vs· never/rarely | -0·03 (-0·04, -0·01) | < 0·001 | < 0·001 |
| Extra salt in food: sometimes vs· never/rarely | -0·02 (-0·03, -0·01) | 0·001 | 0·003 |
| MET category: high vs· low | 0·01 (-0·01, 0·02) | 0·356 | 0·455 |
| MET category: moderate vs· low | -0·01 (-0·03, -0·00) | 0·05 | 0·083 |
| Sleep difficulty: often vs· never/rarely | 0·00 (-0·01, 0·01) | 0·881 | 0·897 |
| Sleep difficulty: sometimes vs· never/rarely | -0·02 (-0·04, -0·01) | < 0·001 | < 0·001 |
| Sleep hours: >9 hours vs· ≤6 hours | 0·01 (-0·01, 0·02) | 0·362 | 0·455 |
| Sleep hours: 6–7 hours vs· ≤6 hours | 0·06 (0·04, 0·07) | < 0·001 | < 0·001 |
| Sleep hours: 7–8 hours vs· ≤6 hours | 0·05 (0·03, 0·07) | < 0·001 | < 0·001 |
| Sleep hours: 8–9 hours vs· ≤6 hours | 0·01 (-0·00, 0·03) | 0·065 | 0·104 |
| Smoking habit: former smoker vs· no | -0·00 (-0·01, 0·01) | 0·432 | 0·496 |
| Smoking habit: yes (both) vs· no | -0·01 (-0·02, 0·00) | 0·084 | 0·125 |
| Smoking habit: yes (conventional cigarettes) vs· no | -0·03 (-0·04, -0·02) | < 0·001 | < 0·001 |
| Smoking habit: yes (e-cigarettes) vs· no | -0·01 (-0·03, 0·00) | 0·07 | 0·11 |
| Snore: yes vs· no | 0·01 (0·00, 0·02) | 0·028 | 0·051 |

***Note*:** CI, confidence interval; MET, metabolic equivalent of task.
