# Supplementary material for: Health equity and public acceptance of large language models in healthcare in China: A national population-based survey
Source: PLOS Digit Health. 2026 Jul 30;5(7):e0001555. doi: 10.1371/journal.pdig.0001555 (PMC13422829; doi:10.1371/journal.pdig.0001555)
Supplement: S10 Table — (DOCX) [file pdig.0001555.s012.docx]

**S10 Table.** Block 6: hierarchical weighted linear regression of physical health predictors on acceptance of large language model in healthcare (n=35,861).

| **Predictor** | **Standardized β (95% CI)** | **p** | **Adjusted p** |
| --- | --- | --- | --- |
| Arthritis diagnosis: yes vs· no | -0·01 (-0·02, 0·00) | 0·108 | 0·171 |
| Coronary heart disease diagnosis: yes vs· no | 0·00 (-0·00, 0·01) | 0·368 | 0·476 |
| COVID-positive count | -0·02 (-0·03, -0·01) | 0·001 | 0·002 |
| Diabetes diagnosis: yes vs· no | 0·00 (-0·01, 0·01) | 0·500 | 0·616 |
| Digestive disease diagnosis: yes vs· no | 0·00 (-0·01, 0·01) | 0·708 | 0·785 |
| EQ-5D index (0·002–0·953) | 0·01 (-0·00, 0·02) | 0·194 | 0·278 |
| Flu vaccination: yes vs· no | -0·01 (-0·02, 0·00) | 0·136 | 0·205 |
| Hepatitis vaccination: yes vs· no | 0·03 (0·01, 0·04) | < 0·001 | < 0·001 |
| HPV vaccination: yes vs· no | 0·02 (0·00, 0·03) | 0·007 | 0·015 |
| Hyperlipidemia diagnosis: yes vs· no | 0·02 (0·01, 0·03) | < 0·001 | < 0·001 |
| Hypertension diagnosis: yes vs· no | 0·01 (0·00, 0·02) | 0·005 | 0·011 |
| Injury event: animal‐related injury: yes vs· no | -0·01 (-0·02, 0·00) | 0·076 | 0·122 |
| Injury event: blunt instrument injury: yes vs· no | 0·00 (-0·01, 0·01) | 0·873 | 0·916 |
| Injury event: burn or scald: yes vs· no | 0·02 (0·01, 0·03) | 0·002 | 0·005 |
| Injury event: drowning: yes vs· no | -0·00 (-0·01, 0·01) | 0·958 | 0·973 |
| Injury event: fall or falling: yes vs· no | 0·00 (-0·01, 0·01) | 0·709 | 0·785 |
| Injury event: firearm injury: yes vs· no | 0·02 (0·00, 0·03) | 0·004 | 0·010 |
| Injury event: motor vehicle accident: yes vs· no | -0·02 (-0·03, -0·01) | 0·003 | 0·007 |
| Injury event: non‐motor vehicle accident: yes vs· no | 0·00 (-0·01, 0·01) | 0·676 | 0·785 |
| Injury event: other: yes vs· no | 0·02 (0·00, 0·03) | 0·003 | 0·007 |
| Injury event: poisoning: yes vs· no | -0·00 (-0·01, 0·01) | 0·772 | 0·818 |
| Injury event: sexual assault: yes vs· no | 0·02 (0·00, 0·03) | 0·037 | 0·064 |
| Injury event: sharp‐object injury: yes vs· no | 0·02 (0·01, 0·04) | < 0·001 | 0·001 |
| Injury event: suffocation or hanging: yes vs· no | -0·02 (-0·03, -0·00) | 0·006 | 0·013 |
| Osteoporosis diagnosis: yes vs· no | -0·01 (-0·02, 0·00) | 0·067 | 0·111 |
| Other diagnosis: yes vs· no | 0·00 (-0·01, 0·01) | 0·711 | 0·785 |
| Rare disease diagnosis: yes vs· no | -0·00 (-0·01, 0·01) | 0·685 | 0·785 |
| Respiratory disease diagnosis: yes vs· no | 0·00 (-0·01, 0·01) | 0·973 | 0·973 |
| Shingles vaccination: yes vs· no | -0·00 (-0·02, 0·01) | 0·735 | 0·803 |
| Stroke diagnosis: yes vs· no | 0·00 (-0·01, 0·01) | 0·610 | 0·719 |
| Tumor diagnosis: yes vs· no | 0·01 (-0·00, 0·02) | 0·164 | 0·238 |
| Urinary disease diagnosis: yes vs· no | -0·00 (-0·01, 0·01) | 0·743 | 0·803 |

***Note***: CI, confidence interval; COVID, coronavirus disease 2019; EQ-5D, EuroQol five-dimension health status index; HPV, human papillomavirus.
