# Supplementary material for: Health equity and public acceptance of large language models in healthcare in China: A national population-based survey
Source: PLOS Digit Health. 2026 Jul 30;5(7):e0001555. doi: 10.1371/journal.pdig.0001555 (PMC13422829; doi:10.1371/journal.pdig.0001555)
Supplement: S11 Table — (DOCX) [file pdig.0001555.s013.docx]

**S11 Table.** Block 7: hierarchical weighted linear regression of mental-health predictors on acceptance of large language model in healthcare (n=35,861).

| **Predictor** | **Standardized β (95% CI)** | **p** | **Adjusted p** |
| --- | --- | --- | --- |
| ASRS-6: ADHD symptoms (0–24) | -0·08 (-0·10, -0·06) | < 0·001 | < 0·001 |
| BSMAS: social media addiction (6–30) | 0·05 (0·04, 0·07) | < 0·001 | < 0·001 |
| CCBI-7: work burnout (7–35) | 0·06 (0·04, 0·08) | < 0·001 | < 0·001 |
| GAD-3: anxiety (0–9) | 0·00 (-0·01, 0·02) | 0·712 | 0·728 |
| MDS5: maladaptive daydreaming (0–100) | -0·04 (-0·05, -0·02) | < 0·001 | < 0·001 |
| Personal existence (1–7) | 0·05 (0·04, 0·07) | < 0·001 | < 0·001 |
| RSS: rest intolerance (8–40) | 0·03 (0·01, 0·04) | 0·001 | 0·003 |
| SCS: cyberchondria (4–20) | 0·04 (0·03, 0·05) | < 0·001 | < 0·001 |

***Note***: CI, confidence interval; ASRS-6, Adult ADHD Self-Report Scale-6 item; BSMAS, Bergen Social Media Addiction Scale; CCBI-7, 7-item work burnout scale; GAD-3, 3-item Generalized Anxiety Disorder scale; MDS5, Maladaptive Daydreaming Scale-5 item; RSS, Rest Intolerance Scale; SCS, Cyberchondria Scale.
