# Supplementary material for: Health equity and public acceptance of large language models in healthcare in China: A national population-based survey
Source: PLOS Digit Health. 2026 Jul 30;5(7):e0001555. doi: 10.1371/journal.pdig.0001555 (PMC13422829; doi:10.1371/journal.pdig.0001555)
Supplement: Attachment — Submitted filename: Peer Review Report PDIG-D-26-00459 R1.pdf [file pdig.0001555.s017.pdf]

Peer Review Report

Health equity and public acceptance of large language models in healthcare in China: a national population-based survey

PLOS Digital Health

PDIG-D-26-00459 R1

Assessment after revision made: The manuscript has been substantially improved following the revisions made and is now recommended for publication.
