# Supplementary material for: Health equity and public acceptance of large language models in healthcare in China: A national population-based survey
Source: PLOS Digit Health. 2026 Jul 30;5(7):e0001555. doi: 10.1371/journal.pdig.0001555 (PMC13422829; doi:10.1371/journal.pdig.0001555)
Supplement: Attachment — Submitted filename: Response to Reviewers.docx [file pdig.0001555.s016.docx]

**Reviewer 1: Peer Review Report**

**Health equity and public acceptance of large language models in healthcare in China: a national population-based survey**

**PLOS Digital Health**

**PDIG-D-26-00459**

Thank you for the opportunity for me to review this research article. The paper deals with an important topic of assessing the public acceptance of large language models in China regarding patients’ and people’s acceptance and readiness to use AI-related digital resources for improvement of their health status while reducing the inequalities of healthcare service/support with digital access. The paper also adopted nation-wide dataset which contains a representative sample of the Chinese population which enhance its validity of generalizability at least in the Chinese context. Regarding the rooms for improvements, I have the following observations.

**Response:** We sincerely thank the reviewer for the kind and encouraging comments. We are grateful for the recognition of the study’s importance, national dataset, and relevance to digital health equity in China. We have carefully addressed the reviewer’s valuable suggestions to further improve the manuscript. In this response letter, our responses are highlighted in purple, and manuscript changes are highlighted in blue to make them easier to track. Thank you once again!

**Comment 1.** First, in p.4, line 80, the authors have indicated a research gap. However, stressing the importance with explanation why particularly investigating LLM is with practical/scientific importance? For example, failure to understand such acceptance is with detrimental outcomes for patients? For practitioners? Or for policy makers?

**Response:** We thank the reviewer for highlighting this critical point. We have added a new concluding section to this paragraph that clearly outlines the detrimental consequences of failing to understand LLM acceptance across three key stakeholders: patients (widening the digital divide), practitioners (workflow inefficiencies), and policymakers (financing structural inequities).

**Changes made in manuscript:** The following blue sentences have been added in the Introduction section:

“However, much of this evidence relates to AI in general rather than LLMs specifically, and population-based evidence on public acceptance of LLM-integrated healthcare remains limited. Because LLMs interact directly with users, public acceptance is a critical determinant of implementation. Failure to understand socially patterned acceptance may widen digital health inequities, reduce effective engagement with LLM-supported services, and lead policymakers to invest in technologies without adequate support for groups less ready to use them.” (Page 4, lines 84-87)

**Comment 2.** Secondly, in p.5, line 91, the authors mentioned the importance to examine the questions. Yet, for understanding the acceptance of the language model, writers should justify their selection of quantitative surveys as methodology rather than other research designs. For example, qualitative interviews and narrative approach also deal with the language features of the AI models or interface designs, why qualitative method is particularly suitable for the subject?

**Response:** We genuinely appreciate this thoughtful comment. We agree that qualitative approaches are valuable for understanding user experience, language interaction, and interface-level mechanisms. As our study aimed to estimate population-level acceptance and identify socially patterned correlates and subgroup profiles, we used a large quantitative population-based survey. We have revised the Introduction to clarify this rationale, while acknowledging the continued importance of qualitative and design-focused studies for understanding mechanisms and improving implementation.

**Changes made in manuscript:** The following blue sentences have been added in Introduction section:

**“**China provides an important setting in which to examine these questions. As one of the world’s most rapidly digitizing health environments, it offers a valuable context for assessing public acceptance of LLMs at scale and for identifying groups who may be less likely to engage with these technologies. While qualitative approaches are highly valuable for unpacking the nuances of human-computer interaction and interface preferences, quantifying the systemic digital divide requires population-level epidemiological profiling. Therefore, a large-scale, quantitative survey is methodologically necessary to systematically capture the distribution of acceptance and mathematically model the structural, social, and psychological determinants across a massive, heterogeneous population.” (Page 5, lines 101-106)

**Comment 3.** Thirdly, in p.5, line 106, before sampling stages. The author should explain how the respondents were recruited, e.g., by convenience email or reaching out for participants?

**Response:** Thank you for this helpful comment. We have supplemented the recruitment methods. Specifically, trained surveyors conducted field-based recruitment at selected survey sites, screened eligibility, obtained electronic informed consent, and administered the electronic questionnaire.

**Changes made in manuscript:** The following blue sentences have been added in the Methods section:

**“2. Methods**

**2.1 Study design and participants**

This study is a secondary analysis of data from a national cross-sectional survey in China that was originally designed to assess the psychological and behavioral health. The survey used a multi-stage stratified sampling strategy. Participants were recruited through field-based on-site recruitment. Trained surveyors visited selected survey sites, screened potential participants for eligibility, obtained electronic informed consent, and administered the electronic questionnaire. To ensure representativeness, we selected 150 cities ...” (Page 6, lines 118-120)

**Comment 4.** Fourthly, in p.6, line 117, the authors should give details how rights of the respondents, including voluntary participation and confidentiality principles were complied throughout the study, despite the ethical approval was included by the end of the paper.

**Response:** Thank you for this important suggestion. We have added details on participant rights and confidentiality in the Methods. We clarified that participation was voluntary, informed consent was obtained before data collection, respondents could decline participation, and data were de-identified and analyzed in aggregate form.

**Changes made in manuscript:** The following blue sentence have been added:

“**2.2 Data collection**

Between 23 June and 29 September 2024, trained surveyors conducted on-site recruitment and administered an electronic questionnaire. Prior to enrollment, surveyors detailed the study’s purpose, procedures, and the voluntary nature of participation, ensuring respondents understood their right to decline. All participants provided explicit electronic informed consent. To protect confidentiality, survey data were securely stored, de-identified prior to analysis, and reported exclusively in aggregate. To ensure data quality, responses underwent logic verification and automated quality ...” (Page 6, lines 129-132)

**Comment 5.** Also, in p.15, the Panel A shows a bounce back effect as those above age 90 demonstrate better acceptance over those seniors at a younger age. This pattern is intriguing and warrants further discussion. The author might consider whether this is due to a survivorship bias, where individuals reaching such an advanced age possess higher-than-average adaptability or digital literacy that contributed to their longevity.

**Response:** We sincerely thank the reviewer for this thoughtful observation and insightful interpretation. We agree that the apparent bounce back in the ≥90 age group is interesting and may reflect survivorship bias, whereby very old adults represent a particularly resilient subgroup. At the same time, we acknowledge that this estimate should be interpreted cautiously because the ≥90 group had a small sample size and wide confidence interval. We have revised the Discussion to mention both this possible explanation and the related statistical uncertainty.

**Changes made in manuscript:** The following blue sentence have been revised in the Discussion section:

“... Conversely, adults aged ≥90 years exhibited an apparent rebound in acceptance. While this pattern may reflect survivorship bias, with nonagenarians representing a select and potentially more resilient subgroup, the exceptionally wide confidence intervals in this stratum indicate substantial statistical uncertainty. Future research is needed to determine whether this pattern reflects a true cohort-related difference or random sampling variability. ...” (Page 25, lines 353-357)

**Comment 6.** Moreover, in p.24, line 315, there are literature detailing elderly’s reluctance to accept digital resource owing to traditional Chinese culture, e.g., their preference for “simple late lives”, and their digital-related expense’s violation with Chinese’s tenant regarding frugality. The author should consider providing such explanations.

**Response:** We thank the reviewer for this excellent and nuanced suggestion. We fully agree that the digital divide among older adults in China cannot be explained by age or technological literacy alone; it is deeply rooted in specific socio-cultural values and resource constraints. To address this, we have expanded our Discussion to explicitly incorporate these socio-cultural explanations.

**Changes made in manuscript:** The following blue sentences have been revised accordingly in the Discussion section:

“... In contrast, consistent with broader digital health trends, acceptance declined with age. In China, this reluctance may be compounded by sociocultural and resource barriers: studies of Chinese older adults suggest that digital inclusion is shaped by life-course disadvantage, traditional social roles, limited economic resources, and concerns about cost or device damage, including a culture of thrift that may discourage exploratory technology use[19, 20]. ...” (Page 25, lines 349-353)

**Comment 7.** Besides, in p.25, line 339, a minor coherence point is detected. The author mentioned “to mitigate these inequalities”, yet the discussion on dealing with the low-literacy population in previous sentences are related to the survivors of active threats and of emotional neglects. Please add the discussion about inequalities manifested in low-literacy groups prior to the suggestions.

**Response:** Thank you for this helpful comment. We agree that the previous wording created a coherence gap. We have revised the paragraph to first discuss literacy-related inequality before presenting recommendations.

**Changes made in manuscript:** The following sentence marked in blue have been added:

“... In addition to trauma-related differences, literacy-related disparities were also observed: lower eHealth and general health literacy were associated with lower acceptance of LLMs in healthcare. This suggests that text-heavy or conceptually complex LLM-based systems may disproportionately exclude individuals who have greater difficulty accessing, appraising, and using digital health information. To mitigate these inequalities, ...” (Page 27, lines 389-392)

**Comment 8.** Additionally, in p.26, line 363, another minor point is detected. The suggestion of lowering barriers for digitally excluded is not aligned well with the problem of risk-behavioral takers (e.g., smokers). I suggest the author shift the focus from 'lowering barriers' for an assumed 'excluded' group toward enhancing the perceived utility of the AI for these users. Specifically, the author could recommend program designs that offer real-time, actionable support—such as AI-driven salt/calorie trackers or non-judgmental 'nudges' for behavioral change—which may better address the motivational barriers to acceptance in these populations

**Response:** We greatly appreciate this insightful conceptual pivot. We have revised the conclusion of this paragraph to align with your excellent suggestion, shifting the focus from "lowering barriers" to "enhancing utility" through actionable, non-judgmental AI behavioral

nudges.

**Changes made in manuscript:** the following blue sentences have been added:

“... likely reflects a broader profile often associated with lower SES[32]. To engage populations with specific risk profiles, implementation strategies must shift from merely lowering access barriers to actively enhancing the technology’s perceived utility. Future LLM-enabled healthcare tools should integrate real-time, actionable support, such as conversational dietary trackers or personalized, non-judgmental nudges for behavioral change, to overcome these groups' distinct motivational barriers to acceptance.” (Page 28, lines 419-424)

Finally, we sincerely thank the reviewer for their time, expertise, and helpful comments, which have greatly helped us improve our work. Thank you once again.

**Reviewer 2: REVIEWER’S COMMENT**

Manuscript Title: Health Equity And Public Acceptance Of Large Language Models In Healthcare In China: A National Population-Based Survey

Journal: PLOS Digital Health

# Summary of the Manuscript

The manuscript presents a large, nationally representative survey examining public acceptance of large language models (LLMs) in healthcare across China. Using a multistage stratified sample of 35,861 adults, the study assesses acceptance levels and explores a wide range of bio-psycho-social correlates through survey-weighted regression and classification tree analysis. The study identifies moderate overall acceptance, with significant variation across population subgroups, and highlights potential inequities in the adoption of AI-enabled healthcare tools.

# Overall Assessment

The topic in digital health, particularly the intersection of AI and health equity addressed in this manuscript is highly relevant and timely. Notable strengths include its large sample size, national scope, and comprehensive analytical approach. The study aligns well with the aims of PLOS Digital Health and has clear potential to contribute to the field. However, there are several conceptual, methodological, and interpretative issues that should be addressed to strengthen the rigor and clarity of the work before publication.

**Response:** We sincerely thank the reviewer for the thoughtful summary and encouraging overall assessment. We are grateful for the recognition of our study and appreciate the reviewer’s constructive comments. We have carefully revised the manuscript to improve its clarity, rigor, and balance, and we provide detailed point-by-point responses below. In this response letter, our responses are highlighted in purple, and manuscript changes are highlighted in blue to make them easier to track. Thank you once again.

# Major Comments

**Comment 1.** Conceptualization of 'acceptance'. The conceptual definition of 'acceptance' of LLMs in healthcare could be made clearer to benefit the manuscript. It is not clear whether the construct of ‘acceptance’ reflects trust, willingness to use, perceived usefulness, or general attitudes.

**Response:** Thank you for this insightful comment. We revised the Introduction to define “acceptance” as self-reported willingness or readiness to use LLMs in healthcare after a standardized description, and clarified that it is distinct from trust, perceived usefulness, and general attitudes.

**Changes made in manuscript:** The following blue sentences have been added in the Introduction section:

“Public acceptance matters because LLMs are increasingly positioned as direct or semi-direct interfaces between individuals and health information or services. In this study, we define acceptance as self-reported willingness or readiness to use LLMs in healthcare after receiving a standardized description. This construct is distinct from trust, perceived usefulness, and general attitudes, and reflects a proximal implementation outcome indicating public readiness to engage with LLM-enabled healthcare. ... ” (Page 4, lines 72-76)

**Comment 2.** Measurement validity. Using a single 0–100 scale raises concerns about construct validity especially, following a standardized description. It may reflect hypothetical attitudes rather than real-world behavior particularly given varying levels of familiarity with LLMs.

**Response:** We sincerely appreciate this methodological critique. We agree that observed, real-world behavior is the gold standard. Because direct patient-facing LLM applications are still largely in the developmental or early-rollout phases globally, population-level real-world usage data does not yet exist. Therefore, we measured behavioral intention (willing to use). To reduce respondent burden in a large survey, we used a 0-100 visual analog scale and provided a standardized description to ensure a basic shared understanding of LLMs before assessment. We have added the exact vignette text to Section 2.3 for transparency and acknowledged the limitation of measuring hypothetical intention rather than actual behavior in the Discussion.

**Changes made in manuscript:** The following blue sentences have been added in the Methods section:

**“2.3 Measures of acceptance of LLMs in healthcare**

The primary outcome was acceptance of LLMs in healthcare, measured on a visual analog scale from 0 (completely unacceptable) to 100 (completely acceptable). To reduce variation in baseline understanding of emerging AI technologies, all participants were presented with a standardized plain-language description of LLMs before rating their acceptance. The standardized text stated: “Large language models (LLMs) are a type of deep learning model trained on massive amounts of text data to generate human-like responses. In healthcare, LLMs can affect patients’ health by providing answers to medical questions, assisting with diagnosis and treatment plans, and automating healthcare administrative tasks.” Following this description, participants were asked: “What is your acceptance of the application of large language models in healthcare?” ” (Page 7, lines 140-147)
The following blue sentences have been added in the Limitation section:

“... Second, acceptance was measured using a single self-report item after a standardized description of LLMs. Therefore, it should be interpreted as hypothetical stated acceptance or readiness, rather than as a validated multidimensional measure of trust, perceived usefulness, intention to use, or real-world adoption. Prior familiarity with LLMs may also have influenced responses. Future studies should use multidimensional scales, behavioral data, and qualitative methods to validate and extend these findings. ... ” (Page 31, lines 484-489)

**Comment 3.** Interpretation of associations. The study’s cross-sectional design limits causal interpretation. Therefore, discussion should avoid the use of causal language which makes it difficult to establish conclusions with a cross-sectional, self-reported study design. Findings may be interpreted as associations instead.

**Response:** Thank you for this important comment. We revised the Discussion to avoid causal language and frame the findings as associations, correlates, and hypothesis-generating patterns.

**Changes made in manuscript:** Relevant causal wording in the Discussion has been revised and

highlighted in blue.

**Comment 4.** Model complexity. The inclusion of many predictors raises concerns about interpretability. Authors should make clearer justification.

**Response:** Thank you for this important comment. We agree that the inclusion of many predictors could reduce interpretability if not clearly justified. We have revised the Methods to clarify that the 139 predictors were selected a priori within a broad biopsychosocial framework, rather than through unrestricted data-driven variable discovery. We also clarified the structured analytic strategy used to manage model complexity, including seven prespecified conceptual blocks, VIF screening, FDR correction, Elastic-Net comparison, and cautious domain-level interpretation.

**Changes made in manuscript:** The following blue sentences have been revised accordingly in the Methods section:

**“**Second, to isolate independent associations while managing high dimensionality, we compared two modeling approaches: survey-weighted hierarchical linear regression and Elastic-Net regularization. Because acceptance of LLMs in healthcare may be associated with multiple social, developmental, psychological, behavioral, and health-related factors, we selected 139 candidate predictors a priori within a broad biopsychosocial framework. To improve interpretability and reduce the risk of overfitting, predictors were organized into seven prespecified conceptual blocks rather than entered as an unstructured set of variables. We entered predictors in seven blocks following a prespecified conceptual order: sociodemographics, adversity, personality and self-efficacy, literacy, empowerment, and social capital, lifestyle, physical health, and mental health. Within each block, we applied Benjamini-Hochberg correction to control the false discovery rate, carrying forward only significant terms to subsequent blocks to reduce model size and limit overinterpretation of unstable associations. Multicollinearity was monitored (variance inflation factor [VIF] cutoff >3)[15]. Then, to validate the hierarchical selection, we fit an Elastic-Net model with all 139 candidate predictors (standardized), with 10-fold cross-validation (α=0.50) to identify the optimal penalty parameter (λ_min_) that minimized the mean squared error. We reported overlap statistics to quantify concordance between the hierarchical and regularized models. Individual coefficients were interpreted cautiously, with emphasis on broader domain-level patterns rather than isolated causal effects.” (Page 9, lines 197-198; page 10, lines 199-212)

**Comment 5.** CART model performance. The CART model shows modest discrimination and low sensitivity, limiting practical usefulness. The reported model performance (AUC 0.62, sensitivity 0.19) suggests limited predictive utility. Authors should more explicitly acknowledged this.

**Response:** Thank you for this important comment. We agree that the CART model had limited predictive utility, particularly given its modest AUC and low sensitivity. We have revised the Discussion to acknowledge this more explicitly. Specifically, we clarified that the model should not be interpreted as a strong individual-level prediction tool. Because acceptance was the predicted outcome, the low sensitivity indicates that the tree captured only a small proportion of acceptors, although its high specificity suggests better identification of non-acceptors. We now frame the CART analysis as an exploratory and descriptive subgroup-profiling tool rather than a model for determining individual readiness or allocating services.

**Changes made in manuscript:** The following sentences have been added in the Discussion section:

“However, the tree should be interpreted carefully. Its discrimination was modest, with a weighted AUC of 0.62 and 68% accuracy, and its low sensitivity indicates limited predictive utility. Because acceptance was the predicted outcome, the low sensitivity indicates that the model captured only a small proportion of acceptors, whereas the high specificity suggests that it was better at identifying non-acceptors. Thus, the apparent accuracy should not be overinterpreted, as it may partly reflect the predominance of non-acceptance profiles. Accordingly, it should not be considered a strong predictive model of individual acceptance. Rather, its main value lies in providing an accessible, descriptive framework for identifying combinations of characteristics associated with acceptance and non-acceptance. From an implementation perspective, this may help generate hypotheses about population subgroups that may require additional engagement, communication, or support, but the tree should not be used alone to determine individual readiness for LLM-enabled healthcare or to guide resource allocation.” (Page 29, lines 433-444)

**Comment 6.** Equity framing. The concept of a 'Digital Matthew Effect' as predicted in the study is compelling but may overextend the findings. It would be helpful to distinguish between structural inequities and individual-level correlates more clearly.

**Response:** Thank you for this important comment. We agree that our original wording may have overextended the findings by implying that the study directly demonstrated a structural “Digital Matthew Effect.” We have revised the Discussion to frame this as a potential equity risk rather than a definitive conclusion. We now distinguish between broader structural contexts, such as digital infrastructure and healthcare access, and individual-level correlates. We also softened causal wording throughout this paragraph.

**Changes made in manuscript:** The following content marked in blue has been revised accordingly:

“Our findings suggest a pattern consistent with a potential “Digital Matthew Effect,” in which people with greater social, economic, and digital resources may be more ready to accept LLM-enabled healthcare. At the structural level, unequal digital infrastructure, healthcare access, and technology exposure may shape opportunities to engage with LLM-enabled healthcare. In our study, urban residence may partly reflect these broader structural contexts[21], but it was measured as an individual-level residential characteristic rather than as a direct measure of infrastructure or service availability. At the individual level, psychosocial and economic markers, including higher perceived social status and advantaged childhood socioeconomic position, were associated with higher acceptance, whereas financial strain and religious affiliation were associated with lower acceptance. Among these factors, religion and age have also been reported in prior digital acceptance studies [18, 22]. While these findings do not prove that LLM-enabled healthcare will inevitably exacerbate health disparities, they highlight a potential risk: without equity-oriented implementation, early adoption by advantaged groups may reproduce existing inequalities in digital health access and benefit.” (Page 26, lines 370-380; Page 27, lines 381-383)

**Comment 7.** Policy implications. The discussion could be strengthened by providing more concrete and actionable equity-oriented implementation strategies.

**Response:** Thank you for this helpful suggestion. We have revised the Implications section to specify equity-oriented implementation strategies at the levels of public communication, assisted access, interface design, preservation of human support, and post-deployment equity monitoring.

**Changes made in manuscript:** The following blue sentences have been added accordingly:

**4.4 Implications for equitable implementation**

Our findings have implications for how LLM-enabled healthcare are introduced into healthcare systems. First, moderate average acceptance should not be taken to imply broad readiness across all subgroups. The observed gradients by age, chronic illness, social position, digital experience, and psychosocial context suggest that uptake may be uneven if implementation relies on passive diffusion alone. Therefore, LLM-enabled healthcare should not be deployed only as a universal digital service and assumed to diffuse equally across the population.

Second, implementation strategies may need to go beyond universal availability and consider targeted approaches for groups less likely to engage. These could include clearer explanations of what LLM-based tools can and cannot do, interfaces that reduce literacy or cognitive burden, and deployment models that preserve human support rather than replacing it. At the communication level, public-facing materials should explain LLM functions, limitations, privacy protections, and when users should seek professional care, using plain language and concrete examples. At the access level, assisted onboarding could be provided through community health centers, primary care clinics, older adult services, or family/caregiver-supported settings, especially for older adults, people with chronic conditions, and those without prior digital health use. At the interface level, tools should include voice-first options, simplified navigation, low-literacy explanations, and clear escalation pathways to human professionals. These findings are also relevant to the implementation of LLM-enabled healthcare in low-resource settings, where barriers may include limited digital infrastructure, low eHealth literacy, affordability concerns, and weaker access to professional support. In such settings, LLM-enabled tools may be more equitable if delivered through low-bandwidth platforms, shared community access points, primary care or community-health-worker support, and voice-based or local-language interfaces rather than standalone text-heavy applications.

Third, evaluation of LLM-enabled services should include equity-sensitive outcomes, including who adopts these tools, who does not, and whether their use narrows or widens existing disparities in access and benefit. Such monitoring should be stratified by age, socioeconomic position, chronic disease status, literacy, social support, and prior digital health use. In addition to uptake, evaluations should track discontinuation, unmet support needs, safety concerns, user trust, and whether clinical or informational benefits are equitably distributed. These steps would help ensure that LLM-enabled healthcare improves access rather than reinforcing existing digital and health inequities.” (Page 30, lines 450-468; Page 31, lines 470-476)

# Minor Comments

**Comment 8.** Clarify the standardize description of LLM capabilities provided to participants

**Response:** Thank you for this helpful comment. We have revised the Methods to provide the standardized description of LLMs and their healthcare applications that was shown to participants before they rated acceptance.

**Changes made in manuscript:** The following content marked in blue has been added:

“To reduce variation in baseline understanding of emerging AI technologies, all participants were presented with a standardized plain-language description of LLMs before rating their acceptance. The standardized text stated: “Large language models (LLMs) are a type of deep learning model trained on massive amounts of text data to generate human-like responses. In healthcare, LLMs can affect patients’ health by providing answers to medical questions, assisting with diagnosis and treatment plans, and automating healthcare administrative tasks.” Following this description, participants were asked: “What is your acceptance of the application of large language models in healthcare?”.” (Page 7, lines 140-147)

**Comment 9.** Ensure consistent terminology (e.g., LLM vs AI tools)

**Response:** Thank you for noting this issue. We now use “LLMs in healthcare” or “LLM-enabled healthcare” when referring to the specific technology studied. We use broader terms such as “AI” only when discussing the wider literature or general digital health context.

**Changes made in manuscript:** Relevant terminology has been revised and highlighted in blue where necessary.

**Comment 10.** Improve figure captions

**Response:** Thank you for this suggestion. We have revised the figure captions to be more self-contained and clear.

**Changes made in manuscript:** The figure captions now have been revised as follows:

**“Figure 1.** Demographic and clinical stratification of survey-weighted mean acceptance of large language models (LLMs) in healthcare (n = 35,861). (A) Weighted mean acceptance by sex and age group. (B) Age-related trends in weighted mean acceptance by sex, visualized using LOESS smoothing. (C) Weighted mean acceptance among respondents with specific chronic conditions compared with the overall population average.

**Figure 2.** Classification decision tree model of survey-weighted acceptance of large language models (LLMs) in healthcare. The model was fitted to the training set (unweighted n=23,940; weighted n=24,316).”

**Comment 11.** Review minor grammatical and formatting inconsistencies

**Response:** Thank you for this comment. We have carefully reviewed the manuscript and corrected minor grammatical, terminology, and formatting inconsistencies.

**Changes made in manuscript:** We corrected minor grammatical and formatting issues throughout the manuscript, where changes are marked in blue.

# Final Suggestion

**Comment 12.** The authors may consider discussing how these findings could inform implementation strategies for AI tools in low-resource settings.

**Response:** Thank you for this helpful suggestion. We have revised the Implications section to discuss how our findings may inform implementation of AI tools in low-resource settings.

**Changes made in manuscript:** The following blue sentences have been added accordingly:

“... These findings are also relevant to the implementation of LLM-enabled healthcare in low-resource settings, where barriers may include limited digital infrastructure, low eHealth literacy, affordability concerns, and weaker access to professional support. In such settings, LLM-enabled tools may be more equitable if delivered through low-bandwidth platforms, shared community access points, primary care or community-health-worker support, and voice-based or local-language interfaces rather than standalone text-heavy applications. ...” (Page 30, lines 462-468)

Finally, we sincerely thank the reviewer for their time, expertise, and helpful comments, which have greatly helped us improve our work. Thank you once again.
