# Supplementary material for: Health equity and public acceptance of large language models in healthcare in China: A national population-based survey
Source: PLOS Digit Health. 2026 Jul 30;5(7):e0001555. doi: 10.1371/journal.pdig.0001555 (PMC13422829; doi:10.1371/journal.pdig.0001555)
Supplement: Attachment — Submitted filename: Reviewer report PDIG-D-26-00459.docx [file pdig.0001555.s014.docx]

**Peer Review Report**

**Health equity and public acceptance of large language models in healthcare in China: a national population-based survey**

**PLOS Digital Health**

**PDIG-D-26-00459**

Thank you for the opportunity for me to review this research article. The paper deals with an important topic of assessing the public acceptance of large language models in China regarding patients’ and people’s acceptance and readiness to use AI-related digital resources for improvement of their health status while reducing the inequalities of healthcare service/support with digital access. The paper also adopted nation-wide dataset which contains a representative sample of the Chinese population which enhance its validity of generalizability at least in the Chinese context. Regarding the rooms for improvements, I have the following observations.

1. First, in p.4, line 80, the authors have indicated a research gap. However, stressing the importance with explanation why particularly investigating LLM is with practical/scientific importance? For example, failure to understand such acceptance is with detrimental outcomes for patients? For practitioners? Or for policy makers?

2. Secondly, in p.5, line 91, the authors mentioned the importance to examine the questions. Yet, for understanding the acceptance of the language model, writers should justify their selection of quantitative surveys as methodology rather than other research designs. For example, qualitative interviews and narrative approach also deal with the language features of the AI models or interface designs, why qualitative method is particularly suitable for the subject?

3. Thirdly, in p.5, line 106, before sampling stages. The author should explain how the respondents were recruited, e.g., by convenience email or reaching out for participants?

4. Fourthly, in p.6, line 117, the authors should give details how rights of the respondents, including voluntary participation and confidentiality principles were complied throughout the study, despite the ethical approval was included by the end of the paper.

5. Also, in p.15, the Panel A shows a bounce back effect as those above age 90 demonstrate better acceptance over those seniors at a younger age. This pattern is intriguing and warrants further discussion. The author might consider whether this is due to a survivorship bias, where individuals reaching such an advanced age possess higher-than-average adaptability or digital literacy that contributed to their longevity.

6. Moreover, in p.24, line 315, there are literature detailing elderly’s reluctance to accept digital resource owing to traditional Chinese culture, e.g., their preference for “simple late lives”, and their digital-related expense’s violation with Chinese’s tenant regarding frugality. The author should consider providing such explanations.

7. Besides, in p.25, line 339, a minor coherence point is detected. The author mentioned “to mitigate these inequalities”, yet the discussion on dealing with the low-literacy population in previous sentences are related to the survivors of active threats and of emotional neglects. Please add the discussion about inequalities manifested in low-literacy groups prior to the suggestions.

8. Additionally, in p.26, line 363, another minor point is detected. The suggestion of lowering barriers for digitally excluded is not aligned well with the problem of risk-behavioral takers (e.g., smokers). I suggest the author shift the focus from 'lowering barriers' for an assumed 'excluded' group toward enhancing the perceived utility of the AI for these users. Specifically, the author could recommend program designs that offer real-time, actionable support—such as AI-driven salt/calorie trackers or non-judgmental 'nudges' for behavioral change—which may better address the motivational barriers to acceptance in these populations
