# Supplementary material for: Health equity and public acceptance of large language models in healthcare in China: A national population-based survey
Source: PLOS Digit Health. 2026 Jul 30;5(7):e0001555. doi: 10.1371/journal.pdig.0001555 (PMC13422829; doi:10.1371/journal.pdig.0001555)
Supplement: Attachment — Submitted filename: Peer_Review_PDIG-D-26-00459.docx [file pdig.0001555.s015.docx]

**REVIEWER’S COMMENT**

Manuscript Title: Health Equity And Public Acceptance Of Large Language Models In Healthcare In China: A National Population-Based Survey

Journal: PLOS Digital Health

# 1. Summary of the Manuscript

Title: Health Equity And Public Acceptance Of Large Language Models In Healthcare In China: A National Population-Based Survey

The manuscript presents a large, nationally representative survey examining public acceptance of large language models (LLMs) in healthcare across China. Using a multistage stratified sample of 35,861 adults, the study assesses acceptance levels and explores a wide range of bio-psycho-social correlates through survey-weighted regression and classification tree analysis. The study identifies moderate overall acceptance, with significant variation across population subgroups, and highlights potential inequities in the adoption of AI-enabled healthcare tools.

# 2. Overall Assessment

The topic in digital health, particularly the intersection of AI and health equity addressed in this manuscript is highly relevant and timely. Notable strengths include its large sample size, national scope, and comprehensive analytical approach. The study aligns well with the aims of PLOS Digital Health and has clear potential to contribute to the field. However, there are several conceptual, methodological, and interpretative issues that should be addressed to strengthen the rigor and clarity of the work before publication.

# 3. Major Comments

1. Conceptualization of 'acceptance'.

The conceptual definition of 'acceptance' of LLMs in healthcare could be made clearer to benefit the manuscript. It is not clear whether the construct of ‘acceptance’ reflects trust, willingness to use, perceived usefulness, or general attitudes.

2. Measurement validity

Using a single 0–100 scale raises concerns about construct validity especially, following a standardized description. It may reflect hypothetical attitudes rather than real-world behavior particularly given varying levels of familiarity with LLMs.

3. Interpretation of associations

The study’s cross-sectional design limits causal interpretation. Therefore, discussion should avoid the use of causal language which makes it difficult to establish conclusions with a cross-sectional, self-reported study design. Findings may be interpreted as associations instead.

4. Model complexity

The inclusion of many predictors raises concerns about interpretability. Authors should make clearer justification.

5. CART model performance

The CART model shows modest discrimination and low sensitivity, limiting practical usefulness. The reported model performance (AUC 0.62, sensitivity 0.19) suggests limited predictive utility. Authors should more explicitly acknowledged this.

6. Equity framing

The concept of a 'Digital Matthew Effect' as predicted in the study is compelling but may overextend the findings. It would be helpful to distinguish between structural inequities and individual-level correlates more clearly.

7. Policy implications

The discussion could be strengthened by providing more concrete and actionable equity-oriented implementation strategies.

# 4. Minor Comments

- Clarify the standardize description of LLM capabilities provided to participants
- Ensure consistent terminology (e.g., LLM vs AI tools)
- Improve figure captions
- Review minor grammatical and formatting inconsistencies

# 5. Evaluation

- Originality: Strong
- Importance: High
- Rigor: Good with minor concerns
- Evidence: Generally supported
- Utility: Good but can improve
- Open science: Moderate

# 6. Recommendation

Major Revision

# 7. Final Suggestion

The authors may consider discussing how these findings could inform implementation strategies for AI tools in low-resource settings.
