# Supplementary material for: Health equity and public acceptance of large language models in healthcare in China: A national population-based survey
Source: PLOS Digit Health. 2026 Jul 30;5(7):e0001555. doi: 10.1371/journal.pdig.0001555 (PMC13422829; doi:10.1371/journal.pdig.0001555)
Supplement: Peer review history

# Peer review: Decision Letter 0

**DOI:** 10.1371/journal.pdig.0001555.r001

**Authors:** Po-Chih Kuo

20 Apr 2026

PDIG-D-26-00459

Health equity and public acceptance of large language models in healthcare in China: a national population-based survey

PLOS Digital Health

Dear Dr. Li,

Thank you for submitting your manuscript to PLOS Digital Health. After careful consideration, we feel that it has merit but does not fully meet PLOS Digital Health's publication criteria as it currently stands. Therefore, we invite you to submit a revised version of the manuscript that addresses the points raised during the review process.

Please submit your revised manuscript by Jun 19 2026 11:59PM. If you will need more time than this to complete your revisions, please reply to this message or contact the journal office at digitalhealth@plos.org. When you're ready to submit your revision, log on to https://www.editorialmanager.com/pdig/ and select the 'Submissions Needing Revision' folder to locate your manuscript file.

Please include the following items when submitting your revised manuscript:

* A letter that responds to each point raised by the editor and reviewer(s). You should upload this letter as a separate file labeled 'Response to Reviewers'. This file does not need to include responses to any formatting updates and technical items listed in the 'Journal Requirements' section below.

* A marked-up copy of your manuscript that highlights changes made to the original version. You should upload this as a separate file labeled 'Revised Manuscript with Track Changes'.

* An unmarked version of your revised paper without tracked changes. You should upload this as a separate file labeled 'Manuscript'.

If you would like to make changes to your financial disclosure, competing interests statement, or data availability statement, please make these updates within the submission form at the time of resubmission. Guidelines for resubmitting your figure files are available below the reviewer comments at the end of this letter.

We look forward to receiving your revised manuscript.

Kind regards,

Po-Chih Kuo, Ph. D.

Section Editor

PLOS Digital Health

Po-Chih Kuo

Section Editor

PLOS Digital Health

Leo Anthony Celi

Editor-in-Chief

PLOS Digital Health

orcid.org/0000-0001-6712-6626

**Journal Requirements:**

1. Please provide separate figure files in .tif or .eps format.

For more information about figure files please see our guidelines:  LINK

https://journals.plos.org/digitalhealth/s/figures

https://journals.plos.org/digitalhealth/s/figures#loc-file-requirements

If the reviewer comments include a recommendation to cite specific previously published works, please review and evaluate these publications to determine whether they are relevant and should be cited. There is no requirement to cite these works unless the editor has indicated otherwise.

**Additional Editor Comments (if provided):**

[Note: HTML markup is below. Please do not edit.]

**Reviewers' Comments:**

Reviewer's Responses to Questions

**Comments to the Author**

1. Does this manuscript meet PLOS Digital Health’s publication criteria? Is the manuscript technically sound, and do the data support the conclusions? The manuscript must describe methodologically and ethically rigorous research with conclusions that are appropriately drawn based on the data presented.

Reviewer #1: Yes

Reviewer #2: Yes

**********

2. Has the statistical analysis been performed appropriately and rigorously?

Reviewer #1: Yes

Reviewer #2: Yes

**********

3. Have the authors made all data underlying the findings in their manuscript fully available (please refer to the Data Availability Statement at the start of the manuscript PDF file)?

The PLOS Data policy requires authors to make all data underlying the findings described in their manuscript fully available without restriction, with rare exception. The data should be provided as part of the manuscript or its supporting information, or deposited to a public repository. For example, in addition to summary statistics, the data points behind means, medians and variance measures should be available. If there are restrictions on publicly sharing data—e.g. participant privacy or use of data from a third party—those must be specified.

Reviewer #1: Yes

Reviewer #2: Yes

**********

4. Is the manuscript presented in an intelligible fashion and written in standard English?

PLOS Digital Health does not copyedit accepted manuscripts, so the language in submitted articles must be clear, correct, and unambiguous. Any typographical or grammatical errors should be corrected at revision, so please note any specific errors here.

Reviewer #1: Yes

Reviewer #2: Yes

**********

5. Review Comments to the Author

Please use the space provided to explain your answers to the questions above. You may also include additional comments for the author, including concerns about dual publication, research ethics, or publication ethics. (Please upload your review as an attachment if it exceeds 20,000 characters)

Reviewer #1: Thank you for the opportunity for me to review this research article. The paper deals with an important topic of assessing the public acceptance of large language models in China regarding patients’ and people’s acceptance and readiness to use AI-related digital resources for improvement of their health status while reducing the inequalities of healthcare service/support with digital access. The paper also adopted nation-wide dataset which contains a representative sample of the Chinese population which enhance its validity of generalizability at least in the Chinese context. Regarding the rooms for improvements, I have the following observations.

1. First, in p.4, line 80, the authors have indicated a research gap. However, stressing the importance with explanation why particularly investigating LLM is with practical/scientific importance? For example, failure to understand such acceptance is with detrimental outcomes for patients? For practitioners? Or for policy makers?

2. Secondly, in p.5, line 91, the authors mentioned the importance to examine the questions. Yet, for understanding the acceptance of the language model, writers should justify their selection of quantitative surveys as methodology rather than other research designs. For example, qualitative interviews and narrative approach also deal with the language features of the AI models or interface designs, why qualitative method is particularly suitable for the subject?

3. Thirdly, in p.5, line 106, before sampling stages. The author should explain how the respondents were recruited, e.g., by convenience email or reaching out for participants?

4. Fourthly, in p.6, line 117, the authors should give details how rights of the respondents, including voluntary participation and confidentiality principles were complied throughout the study, despite the ethical approval was included by the end of the paper.

5. Also, in p.15, the Panel A shows a bounce back effect as those above age 90 demonstrate better acceptance over those seniors at a younger age. This pattern is intriguing and warrants further discussion. The author might consider whether this is due to a survivorship bias, where individuals reaching such an advanced age possess higher-than-average adaptability or digital literacy that contributed to their longevity.

6. Moreover, in p.24, line 315, there are literature detailing elderly’s reluctance to accept digital resource owing to traditional Chinese culture, e.g., their preference for “simple late lives”, and their digital-related expense’s violation with Chinese’s tenant regarding frugality. The author should consider providing such explanations.

7. Besides, in p.25, line 339, a minor coherence point is detected. The author mentioned “to mitigate these inequalities”, yet the discussion on dealing with the low-literacy population in previous sentences are related to the survivors of active threats and of emotional neglects. Please add the discussion about inequalities manifested in low-literacy groups prior to the suggestions.

8. Additionally, in p.26, line 363, another minor point is detected. The suggestion of lowering barriers for digitally excluded is not aligned well with the problem of risk-behavioral takers (e.g., smokers). I suggest the author shift the focus from 'lowering barriers' for an assumed 'excluded' group toward enhancing the perceived utility of the AI for these users. Specifically, the author could recommend program designs that offer real-time, actionable support—such as AI-driven salt/calorie trackers or non-judgmental 'nudges' for behavioral change—which may better address the motivational barriers to acceptance in these populations

Reviewer #2: 1. Summary of the Manuscript

Title: Health Equity And Public Acceptance Of Large Language Models In Healthcare In China: A National Population-Based Survey

The manuscript presents a large, nationally representative survey examining public acceptance of large language models (LLMs) in healthcare across China. Using a multistage stratified sample of 35,861 adults, the study assesses acceptance levels and explores a wide range of bio-psycho-social correlates through survey-weighted regression and classification tree analysis. The study identifies moderate overall acceptance, with significant variation across population subgroups, and highlights potential inequities in the adoption of AI-enabled healthcare tools.

2. Overall Assessment

The topic in digital health, particularly the intersection of AI and health equity addressed in this manuscript is highly relevant and timely. Notable strengths include its large sample size, national scope, and comprehensive analytical approach. The study aligns well with the aims of PLOS Digital Health and has clear potential to contribute to the field. However, there are several conceptual, methodological, and interpretative issues that should be addressed to strengthen the rigor and clarity of the work before publication.

3. Major Comments

1. Conceptualization of 'acceptance'.

The conceptual definition of 'acceptance' of LLMs in healthcare could be made clearer to benefit the manuscript. It is not clear whether the construct of ‘acceptance’ reflects trust, willingness to use, perceived usefulness, or general attitudes.

2. Measurement validity

Using a single 0–100 scale raises concerns about construct validity especially, following a standardized description. It may reflect hypothetical attitudes rather than real-world behavior particularly given varying levels of familiarity with LLMs.

3. Interpretation of associations

The study’s cross-sectional design limits causal interpretation. Therefore, discussion should avoid the use of causal language which makes it difficult to establish conclusions with a cross-sectional, self-reported study design. Findings may be interpreted as associations instead.

4. Model complexity

The inclusion of many predictors raises concerns about interpretability. Authors should make clearer justification.

5. CART model performance

The CART model shows modest discrimination and low sensitivity, limiting practical usefulness. The reported model performance (AUC 0.62, sensitivity 0.19) suggests limited predictive utility. Authors should more explicitly acknowledged this.

6. Equity framing

The concept of a 'Digital Matthew Effect' as predicted in the study is compelling but may overextend the findings. It would be helpful to distinguish between structural inequities and individual-level correlates more clearly.

7. Policy implications

The discussion could be strengthened by providing more concrete and actionable equity-oriented implementation strategies.

4. Minor Comments

• Clarify the standardize description of LLM capabilities provided to participants

• Ensure consistent terminology (e.g., LLM vs AI tools)

• Improve figure captions

• Review minor grammatical and formatting inconsistencies

5. Evaluation

• Originality: Strong

• Importance: High

• Rigor: Good with minor concerns

• Evidence: Generally supported

• Utility: Good but can improve

• Open science: Moderate

6. Recommendation

Major Revision

7. Final Suggestion

The authors may consider discussing how these findings could inform implementation strategies for AI tools in low-resource settings.

**********

6. PLOS authors have the option to publish the peer review history of their article (what does this mean?). If published, this will include your full peer review and any attached files.

**Do you want your identity to be public for this peer review?**  If you choose “no”, your identity will remain anonymous but your review may still be made public.

For information about this choice, including consent withdrawal, please see our Privacy Policy.

Reviewer #1: No

Reviewer #2: No

**********

[NOTE: If reviewer comments were submitted as an attachment file, they will be attached to this email and accessible via the submission site. Please log into your account, locate the manuscript record, and check for the action link "View Attachments". If this link does not appear, there are no attachment files.]

**Figure resubmission:**

While revising your submission, we strongly recommend that you use PLOS’s NAAS tool (https://ngplosjournals.pagemajik.ai/artanalysis) to test your figure files. NAAS can convert your figure files to the TIFF file type and meet basic requirements (such as print size, resolution), or provide you with a report on issues that do not meet our requirements and that NAAS cannot fix.

After uploading your figures to PLOS’s NAAS tool - https://ngplosjournals.pagemajik.ai/artanalysis, NAAS will process the files provided and display the results in the "Uploaded Files" section of the page as the processing is complete. If the uploaded figures meet our requirements (or NAAS is able to fix the files to meet our requirements), the figure will be marked as "fixed" above. If NAAS is unable to fix the files, a red "failed" label will appear above. When NAAS has confirmed that the figure files meet our requirements, please download the file via the download option, and include these NAAS processed figure files when submitting your revised manuscript.

**Reproducibility:**

To enhance the reproducibility of your results, we recommend that authors of applicable studies deposit laboratory protocols in protocols.io, where a protocol can be assigned its own identifier (DOI) such that it can be cited independently in the future. Additionally, PLOS ONE offers an option to publish peer-reviewed clinical study protocols. Read more information on sharing protocols at https://plos.org/protocols?utm_medium=editorial-email&utm_source=authorletters&utm_campaign=protocols

Attachment Submitted filename: Reviewer report PDIG-D-26-00459.docx

Attachment Submitted filename: Peer_Review_PDIG-D-26-00459.docx

# Author comment: Author response to Decision Letter 1

**DOI:** 10.1371/journal.pdig.0001555.r002

14 May 2026

Attachment Submitted filename: Response to Reviewers.docx

# Peer review: Decision Letter 1

**DOI:** 10.1371/journal.pdig.0001555.r003

**Authors:** Po-Chih Kuo

19 Jun 2026

Health equity and public acceptance of large language models in healthcare in China: a national population-based survey

PDIG-D-26-00459R1

Dear Assistant Professor Li,

We are pleased to inform you that your manuscript 'Health equity and public acceptance of large language models in healthcare in China: a national population-based survey' has been provisionally accepted for publication in PLOS Digital Health.

Before your manuscript can be formally accepted you will need to complete some formatting changes, which you will receive in a follow-up email from a member of our team.

Please note that your manuscript will not be scheduled for publication until you have made the required changes, so a swift response is appreciated.

IMPORTANT: The editorial review process is now complete. PLOS will only permit corrections to spelling, formatting or significant scientific errors from this point onwards. Requests for major changes, or any which affect the scientific understanding of your work, will cause delays to the publication date of your manuscript.

If your institution or institutions have a press office, please notify them about your upcoming paper to help maximize its impact. If they'll be preparing press materials, please inform our press team as soon as possible -- no later than 48 hours after receiving the formal acceptance. Your manuscript will remain under strict press embargo until 2 pm Eastern Time on the date of publication. For more information, please contact digitalhealth@plos.org.

Thank you again for supporting Open Access publishing; we are looking forward to publishing your work in PLOS Digital Health.

Best regards,

Po-Chih Kuo, Ph. D.

Section Editor

PLOS Digital Health

***********************************************************

**Additional Editor Comments (if provided):**

**Reviewer Comments (if any, and for reference):**

Reviewer's Responses to Questions

**Comments to the Author**

1. If the authors have adequately addressed your comments raised in a previous round of review and you feel that this manuscript is now acceptable for publication, you may indicate that here to bypass the “Comments to the Author” section, enter your conflict of interest statement in the “Confidential to Editor” section, and submit your "Accept" recommendation.

Reviewer #1: All comments have been addressed

Reviewer #2: All comments have been addressed

**********

2. Does this manuscript meet PLOS Digital Health’s publication criteria? Is the manuscript technically sound, and do the data support the conclusions? The manuscript must describe methodologically and ethically rigorous research with conclusions that are appropriately drawn based on the data presented.

Reviewer #1: Yes

Reviewer #2: Yes

**********

3. Has the statistical analysis been performed appropriately and rigorously?

Reviewer #1: Yes

Reviewer #2: Yes

**********

4. Have the authors made all data underlying the findings in their manuscript fully available (please refer to the Data Availability Statement at the start of the manuscript PDF file)?

The PLOS Data policy requires authors to make all data underlying the findings described in their manuscript fully available without restriction, with rare exception. The data should be provided as part of the manuscript or its supporting information, or deposited to a public repository. For example, in addition to summary statistics, the data points behind means, medians and variance measures should be available. If there are restrictions on publicly sharing data—e.g. participant privacy or use of data from a third party—those must be specified.

Reviewer #1: Yes

Reviewer #2: Yes

**********

5. Is the manuscript presented in an intelligible fashion and written in standard English?

PLOS Digital Health does not copyedit accepted manuscripts, so the language in submitted articles must be clear, correct, and unambiguous. Any typographical or grammatical errors should be corrected at revision, so please note any specific errors here.

Reviewer #1: Yes

Reviewer #2: Yes

**********

6. Review Comments to the Author

Please use the space provided to explain your answers to the questions above. You may also include additional comments for the author, including concerns about dual publication, research ethics, or publication ethics. (Please upload your review as an attachment if it exceeds 20,000 characters)

Reviewer #1: The manuscript has been substantially improved following the revisions made and is now recommended for publication.

Reviewer #2: The authors have responded thoughtfully and comprehensively to the reviewer comments. The manuscript has been substantially strengthened, particularly with respect to conceptual clarity, methodological transparency, interpretation of findings, and practical implications. Most concerns have been satisfactorily addressed. Since the revisions are fully reflected in the manuscript, I have no major remaining concerns.

**********

7. PLOS authors have the option to publish the peer review history of their article (what does this mean?). If published, this will include your full peer review and any attached files.

**Do you want your identity to be public for this peer review?**  If you choose “no”, your identity will remain anonymous but your review may still be made public.

For information about this choice, including consent withdrawal, please see our Privacy Policy.

Reviewer #1: **Yes:** Chi Yuen, Lai

Reviewer #2: No

**********

Attachment Submitted filename: Peer Review Report PDIG-D-26-00459 R1.pdf
